# Prolonged Survival of Neutrophils Induced by Tumor‐Derived G‐CSF/GM‐CSF Promotes Immunosuppression and Progression in Laryngeal Squamous Cell Carcinoma

**DOI:** 10.1002/advs.202400836

**Published:** 2024-10-24

**Authors:** Xiaoke Zhu, Yu Heng, Jingyu Ma, Duo Zhang, Di Tang, Yangyang Ji, Changding He, Hanqing Lin, Xuping Ding, Jian Zhou, Lei Tao, Liming Lu

**Affiliations:** ^1^ Department of Otolaryngology Shanghai Key Clinical Disciplines of otorhinolaryngology Eye Ear Nose & Throat Hospital Fudan University Shanghai 200025 P. R. China; ^2^ Shanghai Institute of Immunology Department of Immunology and Microbiology Shanghai Jiao Tong University School of Medicine Shanghai 200025 P. R. China

**Keywords:** CXCR4, extended lifespan, immunosuppression, laryngeal squamous cell carcinoma, neutrophil

## Abstract

Tumor‐associated neutrophils (TANs) play a crucial role in tumor progression and exhibit prolonged survival. However, the mechanism underlying their extended lifespan and significance in laryngeal squamous cell carcinoma (LSCC) remains unclear. Herein, it is observed that apoptosis of TANs is significantly delayed owing to induction by tumor‐derived G‐CSF and GM‐CSF through the activation of the PI3K‐AKT signaling pathway, upregulation of anti‐apoptotic Mcl‐1 expression, and downregulation of activated Caspase‐3 levels. It is found that prolonged survival of TANs leads to the accumulation of aged CXCR4^+^ neutrophils that exhibit potent immunosuppressive properties and are associated with poor patient prognosis. Furthermore, extended survival promotes the enhanced immunosuppressive function of CD8^+^ T cells by TANs, thereby facilitating the in vitro and in vivo progression and growth of human LSCC tumors. Importantly, this effect could be reversed by blocking G‐CSF and GM‐CSF stimulation of neutrophils. These findings elucidate the pivotal role of pathologically prolonged neutrophil survival in impairing CD8^+^ T cell immunity and suggest targeting it as a potential therapeutic strategy for tumors.

## Graphical abstract

This study elucidates a novel mechanism whereby tumor‐derived G‐CSF and GM‐CSF induce delayed neutrophil apoptosis by activating the PI3K‐AKT signaling pathway. Delayed neutrophil apoptosis can facilitate the accumulation of immunosuppressive CXCR4^+^ neutrophils and impair CD8^+^ T cell immunity. These findings suggest targeting the prolonged lifespan of neutrophils under pathological conditions as a potential therapeutic strategy for tumors.

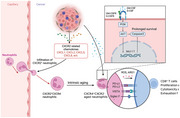

## 1 Introduction

Head and neck squamous cell carcinomas (HNSCC) are malignancies that arise from the mucosal lining of the upper aerodigestive tract, including the oral cavity, larynx, oropharynx, and hypopharynx.^[^ ^1^ ^]^ Laryngeal squamous cell carcinoma (LSCC), one of the most prevalent types of HNSCC, accounts for an estimated 26 400 new cases annually and causes approximately 14500 deaths in China.^[^ ^2^, ^3^, ^4^ ^]^ The aggressive nature of this cancer and its impact on vital functions, such as swallowing, breathing, and speech, make it a significant clinical concern. Despite advancements in multimodal treatment approaches, the 5‐year overall survival rate for patients with advanced LSCC remains suboptimal at approximately 60%.^[^ ^5^ ^]^

Notably, immunotherapy with checkpoint blockade antibodies such as nivolumab and pembrolizumab has demonstrated remarkable efficacy in recurrent or metastatic LSCC; however, these responses have only been observed in a few cases.^[^ ^6^, ^7^ ^]^ The overall response rates to immune checkpoint inhibitors are generally limited and remain below 20% in LSCC.^[^ ^8^, ^9^ ^]^ Multiple interventions aimed at enhancing synthetic and endogenous immune‐mediated tumor cell rejection are currently being extensively investigated,^[^ ^10^ ^]^ but these approaches face significant challenges that necessitate a comprehensive understanding of the intricate interactions between tumor cells and heterogeneous infiltrating immune cells within the tumor microenvironment (TME).

Neutrophils play a crucial role in defending against invading pathogens. They are the most abundant cells in human peripheral blood and are capable of rapid recruitment to sites of inflammation. In the TME, increased neutrophil infiltration has been associated with worse survival outcomes across various cancer types, suggesting that tumor‐associated neutrophils (TANs), or at least certain subsets, broadly contribute to tumor progression.^[^ ^11^, ^12^, ^13^ ^]^ Previous studies have demonstrated that TANs can promote tumor cell proliferation and facilitate angiogenesis through the secretion of soluble factors such as matrix metalloproteinase, prostaglandin E2 (PGE2), and neutrophil elastase (NE).^[^ ^14^, ^15^, ^16^ ^]^ Additionally, they induce immunosuppression by recruiting regulatory T cells into tumors^[^ ^17^ ^]^ and upregulating the expression of immunosuppressive molecules including PD‐L1, PD‐L2, Siglec F, and VISTA.^[^ ^18^, ^19^ ^]^ While most studies support a protumor role of TANs, experimental evidence suggests an incipient anti‐tumor function.^[^ ^20^, ^21^ ^]^ For instance, one study reported that stimulation of a specific subset of TANs expressing the proto‐oncogene MET with hepatocyte growth factor (HGF) resulted in direct killing of tumor cells via nitric oxide release.^[^ ^22^ ^]^ The question regarding heterogeneity among TANs is highly intriguing; however, no molecular evidence or consensus criteria have been reported to definitively identify clinically relevant distinct neutrophil subsets.

Circulating neutrophils are terminally differentiated, non‐proliferative, and have a short lifespan (half‐life of 6‐8 h).^[^ ^23^ ^]^ However, ex vivo data support the hypothesis of prolonged neutrophil survival in vivo under inflammatory conditions and contradict the notion of a short lifespan in vivo.^[^ ^24^, ^25^ ^]^ Recent studies have identified multiple mechanisms contributing to extended neutrophil lifespan, including growth factors and cytokines released by inflammatory cells (e.g., GM‐CSF and IFN‐γ) as well as hypoxia.^[^ ^26^ ^]^ We also observed prolonged neutrophil survival in LSCC^[^ ^18^ ^]^; however, the underlying mechanisms driving this phenomenon and its potential effects on neutrophil heterogeneity and tumor progression remain largely unknown in LSCC.

In this study, we aimed to investigate the mechanism by which LSCC promotes prolonged neutrophil survival while exploring its impact on the suppressive phenotype and heterogeneity of TANs. Additionally, we sought to elucidate the role of these long‐lived neutrophils in the proliferation and exhaustion of CD8^+^ T cells and in tumor progression.

## 2 Results

### 2.1 Increased Infiltration of Neutrophils and Delayed Neutrophil Apoptosis in LSCC

**Figure 1:**
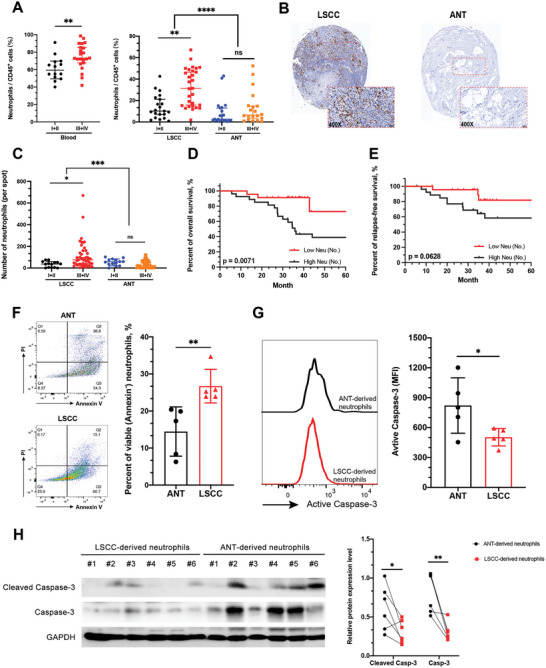
Enhanced infiltration of neutrophils in LSCC correlates with higher tumor stage and unfavorable patient prognosis. A) The proportion of neutrophils expressing CD66b and CD45 markers within the CD45^+^ cell population was analyzed across different tissues in LSCC patients (*n* = 50), with a focus on TNM staging (I+II versus III+IV). B) Representative images of immunohistochemical staining showing the distribution of CD66b^+^ (brown) neutrophils in tissues of LSCC patients. Scale bars: 50 µm. C) The total number of CD66b^+^ (brown) neutrophils stained by immunohistochemistry was analyzed across different tissues in LSCC patients (*n* = 61), with a focus on TNM staging (I+II versus III+IV). Kaplan–Meier curves illustrating the survival rates for D) overall and E) relapse‐free outcomes, categorized based on the median neutrophil count of 61 per spot. F) Representative images of Annexin V/PI staining for neutrophils gating in CD66b^+^CD45^+^ cells. The percentage of viable (Annexin V^−^PI^−^) neutrophils was compared between adjacent normal tissues (*n* = 5) and LSCC tissues (*n* = 5). G) Representative images of active caspase‐3 intracellular staining for neutrophils gating in CD66b^+^CD45^+^ cells. The MFI of active caspase‐3 in neutrophils was compared between adjacent normal tissues (*n* = 5) and LSCC tissues (*n* = 5). H) Level of Caspase‐3 and active caspase‐3 proteins in neutrophils in adjacent normal tissues (*n* = 6) and LSCC tissues (*n* = 6). Statistical analysis was conducted using one‐way ANOVA, Mann‐Whitney U tests, and Student's t‐test (****p < 0.0001, ***p < 0.001, **p < 0.01, *p < 0.05). ns, not significant; LSCC, laryngeal squamous cell carcinoma; ANT, adjacent normal tissue; MFI, mean fluorescence intensity.

To assess the role of neutrophil infiltration in human LSCC, we initially employed flow cytometry to investigate the proportion of CD66b^+^ neutrophils in the total number of CD45^+^ leukocytes in different specimens from patients with LSCC (Cohort 2). Notably, tumor tissues exhibited a significantly higher percentage of infiltrating neutrophils than adjacent normal tissues (ANT). Additionally, as LSCC progressed, both the peripheral blood and tumor tissue displayed a significant increase in the percentage of neutrophils (**Figure** **1**A). Similar findings were observed when analyzing the total number of neutrophils per million cells in each sample (Figure S1A, Supporting Information). Next, immunohistochemistry was used to assess neutrophil infiltration in different tissue specimens from 61 patients with LSCC (Cohort 1). The results also indicated increased accumulation of neutrophils in tumor tissues, which was most noticeable in advanced stage tumors (Figure 1B,C). In line with these results, an increased percentage and number of neutrophils in the tumors were associated with smoking history, advanced tumor stage, increased tumor size, and pathological lymph node fusion (Figures S1B,C and S2, Supporting Information). The results of survival analysis indicated that the 36‐month overall survival and relapse‐free survival rates of patients with high neutrophil number per spot (>50 median level) were lower than that of patients with low neutrophil number per spot (≤50, Figure 1D,E). Results from univariate and multivariate analyses employing Cox proportional hazards models further supported the independent prognostic significance of intratumoral neutrophil count in survival prediction (Table S3, Supporting Information). Collectively, these findings support a correlation between elevated neutrophil infiltration within the LSCC microenvironment and cancer progression, as well as poor prognosis among patients with LSCC.

Unlike other immune cells, circulating neutrophils have a short lifespan and are incapable of dividing.^[^ ^23^ ^]^ To assess variations in lifespan among tissue‐infiltrating neutrophils, Annexin V/PI staining was employed to analyze the proportions of viable and non‐viable neutrophils using flow cytometry. The results revealed a higher percentage of viable (Annexin^−^PI^−^) neutrophils in the tumor tissue than in the ANT (Figure 1F). Caspase‐3 is the primary effector responsible for apoptosis, and activated caspase‐3 can influence key apoptotic features by rapidly cleaving proteins within the cell membrane, cytoskeleton, and nucleus. ^[^ ^27^ ^]^ Intracellular staining showed decreased expression of activated caspase‐3 in LSCC‐derived neutrophils compared to that in ANT‐derived neutrophils (Figure 1G). Comparable findings were noted during the examination of cleaved caspase‐3 and caspase‐3 expression by western blot analysis (Figure 1H). These findings support the hypothesis that the apoptosis of LSCC‐derived neutrophils is significantly delayed.

### 2.2 Tumor Cell‐Derived G‐CSF and GM‐CSF Induce Delayed Neutrophil Apoptosis by Activating the PI3K‐AKT Signaling Pathway

**Figure 2:**
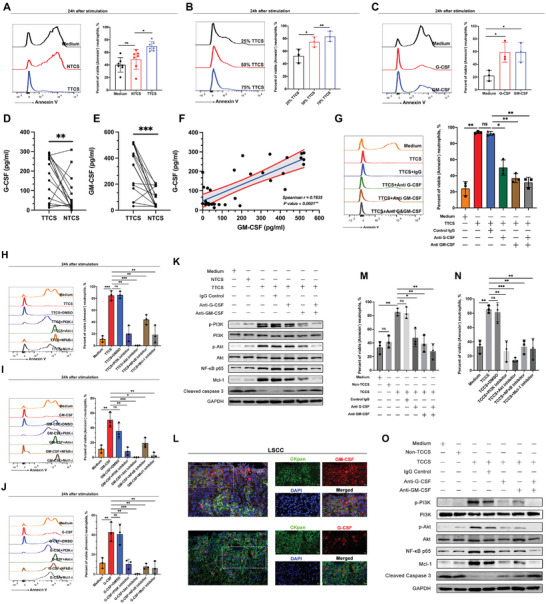
PI3K‐AKT signaling pathway is activated by tumor cell‐derived G‐CSF/GM‐CSF to regulate the delayed apoptosis of neutrophils. A–C) Representative data and quantification of the percentage of viable (Annexin V^−^) cells in neutrophils cultured by 50% autologous NTCS or TTCS for 24 h (A, *n* = 7), or cultured by different concentrations of TTCS for 24 h (B, *n* = 3), or stimulated by G‐CSF (50 ng mL^−1^) or GM‐CSF (50 ng mL^−1^) for 24 h (C, *n* = 3). The concentrations of D) G‐CSF and E) GM‐CSF between autologous NTCS and TTCS (*n* = 16). F)The correlation between G‐CSF and GM‐CSF concentrations. H–J) Representative data and quantification of the percentage of viable (Annexin V^−^) cells in neutrophils cultured by H) 50% TTCS, or stimulated by I) G‐CSF or J) GM‐CSF for 24 h with or without the inhibitors of PI3K, AKT, NFκB, or Mcl‐1 (*n* = 3). K) Levels of various proteins (including PI3K, p‐PI3K, AKT, p‐AKT, NF‐κB p65, Mcl‐1 and cleaved caspase‐3) in neutrophils cultured by 50% autologous NTCS or TTCS with or without antibodies against G‐CSF and/or GM‐CSF. L) Representative images of immunofluorescence staining showing the Ckpan^+^GM‐CSF^+^ or Ckpan^+^G‐CSF^+^ cells within LSCC. Scale bars: 50 µm. Representative data and quantification of the proportion of live (Annexin V^−^) cells in neutrophils cultured by 50% TCCS with or without the presence of antibodies against G‐CSF and/or GM‐CSF for 24 h (M, *n* = 3), or in the presence or absence of the inhibitors of PI3K, AKT, NFκB, or Mcl‐1 (N, *n* = 3). Levels of various proteins (including PI3K, p‐PI3K, AKT, p‐AKT, NF‐κB p65, Mcl‐1 and cleaved caspase‐3) in neutrophils cultured by 50% TCCS with or without the presence of antibodies against G‐CSF and/or GM‐CSF. Statistical analysis was conducted using one‐way ANOVA, Mann‐Whitney U tests, and Student's t‐test (***p < 0.001, **p < 0.01, *p < 0.05). ns, not significant; NTCS, non‐tumor tissue culture supernatant; TTCS, tumor tissue culture supernatant; TCCS, tumor cell culture supernatant; LSCC, laryngeal squamous cell carcinoma.

Neutrophils can be differentially primed by tissue‐derived signals,^[^ ^28^ ^]^ suggesting that tumor‐derived factors may contribute to delayed neutrophil apoptosis. To test this hypothesis, we stimulated neutrophils with tumor tissue culture supernatant (TTCS) or non‐tumor tissue culture supernatant (NTCS) and observed that TTCS significantly prolonged the lifespan of neutrophils compared to NTCS at both the 24 h and 48 h time points (**Figure** **2**A and Figure S3A, Supporting Information). This delay was induced in a dose‐dependent manner (Figure 2B and Figure S3B, Supporting Information). In addition, TTCS contributed to the switch of neutrophils toward immunosuppressive phenotypes, characterized by increased expression of PD‐L1, Siglec F, PD‐L2, and VISTA (Figure S3C, Supporting Information). These results indicate that LSCC environments contribute to delayed neutrophil apoptosis and immunosuppressive phenotypes via the secretion of certain soluble factors such as proinflammatory cytokines. To determine which cytokines might prolong the lifespan of neutrophils, we first screened the genetic expression of proinflammatory cytokines in human head and neck cancer by TCGA analysis (Figure S3D, Supporting Information) and stimulated neutrophils in tumor tissue using differentially or highly expressed cytokines including IL1α, ILβ, IL8, IL33, CXCL5, G‐CSF, M‐CSF, GM‐CSF, TGFβ1, IFNα, CXCL1, and TNFα. The results of Annexin V/PI staining showed that only G‐CSF and GM‐CSF significantly delayed neutrophil apoptosis at both the 24 h and 48 h time points (Figure 2C and Figure S3E–G, Supporting Information). Notably, significantly higher levels of G‐CSF and GM‐CSF were observed in TTCS‐ than in NTCS‐treated cells (Figure 2D,E), with a positive correlation between the production of G‐CSF and GM‐CSF in LSCC (Figure 2F). Notably, efficient inhibition of delayed neutrophil apoptosis was achieved by blocking G‐CSF and/or GM‐CSF in the TTCS/neutrophil co‐culture system (Figure 2G and Figure S3H, Supporting Information). Taken together, these findings indicate that tumor‐derived G‐CSF and GM‐CSF play critical roles in prolonging the lifespan of neutrophils.

Signaling pathway inhibition assays revealed that targeted inhibition of PI3K, AKT, NFκB, or Mcl‐1 signal transduction using specific inhibitors effectively accelerated the apoptosis of TTCS‐conditioned, GM‐CSF‐stimulated, or G‐CSF‐stimulated neutrophils (Figure 2H–J and Figure S4A–E, Supporting Information). Additionally, p‐PI3K and p‐AKT, downstream substrates of the PI3K‐AKT pathway, were significantly upregulated in TTCS‐conditioned neutrophils, which was abrogated by blocking G‐CSF and/or GM‐CSF (Figure 2K). Similar observations were found when analyzing the expression of NFκB and Mcl‐1 (Figure 2K). The expression of cleaved caspase‐3, an indicator of apoptosis, also increased in TTCS‐conditioned neutrophils and decreased after the blockade of G‐CSF and/or GM‐CSF (Figure 2K). These results suggest that the activation of the PI3K‐AKT signaling pathway, upregulation of Mcl‐1, and downregulation of cleaved caspase‐3 are essential for delayed neutrophil apoptosis in the LSCC microenvironment.

Immunofluorescence double‐staining was employed to identify the main source of G‐CSF and GM‐CSF, revealing high expression of these cytokines in LSCC tumors with Ckpan^+^ tumor cells (Figure 2L). Subsequently, Ckpan^+^ cells were isolated from autologous non‐tumor and tumor tissues and cultured to obtain tumor cell culture supernatant (TCCS) and non‐TCCS. Neutrophils were stimulated with the supernatants. Annexin V/PI staining showed that TCCS was superior in prolonging neutrophil lifespan in a dose‐dependent manner compared to non‐TCCS (Figure 2M and Figure S4F,G, Supporting Information). In addition, blockade of G‐CSF and/or GM‐CSF in the TCCS/neutrophil co‐culture system significantly inhibited the delayed apoptosis of neutrophils (Figure 2M and Figure S4G, Supporting Information). Supplementation of the non‐TCCS/neutrophil co‐culture system with human recombinant G‐CSF and/or GM‐CSF efficiently increased the percentage of viable cells (Figure S4H, Supporting Information). Moreover, stimulation with TCCS significantly activated the PI3K‐AKT‐NFκB‐Mcl‐1 signaling pathway while downregulating cleaved caspase‐3 levels in neutrophils, and these conditions were reversed upon blocking G‐CSF and/or GM‐CSF (Figure 2N,O and Figure S4I, Supporting Information). These results collectively demonstrate that tumor cell‐derived G‐CSF/GM‐CSF can delay neutrophil apoptosis by activating the PI3K‐AKT signaling pathway.

### 2.3 Delayed Neutrophil Apoptosis Leads to the Accumulation of Aged CXCR4^+^ Neutrophils in LSCC

**Figure 3:**
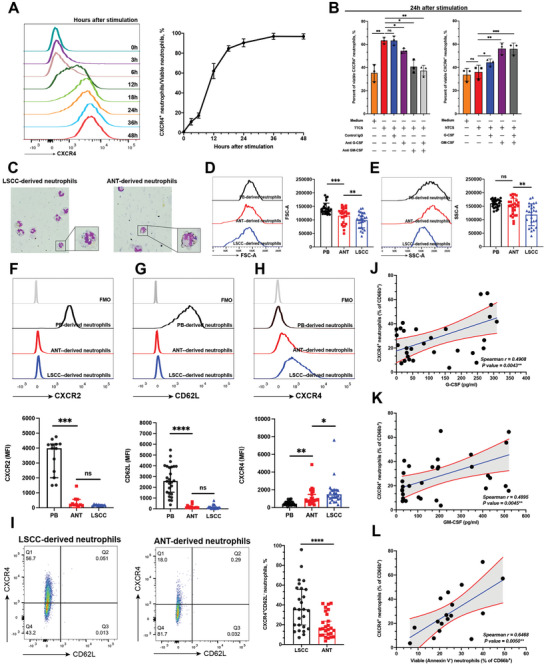
Delayed neutrophil apoptosis promotes the accumulation of CXCR4^+^ aged neutrophils in LSCC. A) The dynamic change of CXCR4 expression at a succession of moments in time on neutrophils cultured by complete RPMI‐1640 medium. B) Representative data and quantification of the percentage of viable (Annexin V^−^) CXCR4^+^ neutrophils cultured by 50% TTCS with or without antibodies against G‐CSF and/or GM‐CSF, or cultured by 50% NTCS with or without human G‐CSF and/or GM‐CSF (*n* = 3). C) Representative images of Giemsa staining showing the nucleus morphology of neutrophils derived from LSCC or ANT. Representative data and quantification of D) FSC‐A and E) SSC‐A of neutrophils derived from PB, ANT or LSCC as measured by flow cytometry (*n* = 26). Representative images of F) CXCR2, G) CD62L, H) CXCR4 staining for CD66b^+^CD45^+^ neutrophils. The MFI of CXCR2 (F, *n* = 13), CD62L (G, *n* = 26), CXCR4 (H, *n* = 26) were compared between neutrophils derived from PB, ANT, and LSCC. I) Representative images and quantification of CXCR4^+^CD62L^−^ aged neutrophils gating in CD66b^+^CD45^+^ cells derived PB, ANT and LSCC (*n* = 26). The correlation between the percentage of CXCR4^+^ neutrophils and the concentration of J) G‐CSF or K) GM‐CSF (K) were analyzed in LSCC tissues. L) The correlation between the percentage of CXCR4^+^ neutrophils and the percentage of viable (Annexin V‐) neutrophils was analyzed in LSCC tissues. Statistical analysis was conducted using one‐way ANOVA, Mann‐Whitney U tests, and Student's t‐test (****p < 0.0001, ***p < 0.001, **p < 0.01, *p < 0.05). ns, not significant; NTCS, non‐tumor tissue culture supernatant; TTCS, tumor tissue culture supernatant; LSCC, laryngeal squamous cell carcinoma; ANT, adjacent normal tissue; PB, peripheral blood; MFI, mean fluorescence intensity.

After mobilization from bone marrow (BM), neutrophils undergo an “aging” process^[^ ^29^ ^]^ in which they downregulate CD62L and upregulate CD11b and CXCR4 before returning to the liver, spleen, and BM for clearance.^[^ ^30^ ^]^ To establish whether delayed neutrophil apoptosis affects the phenotype and function of neutrophils, we first cultured fresh CXCR2^+^CXCR4^−^ neutrophils in complete RPMI‐1640 medium, TTCS, or NTCS and monitored the dynamic changes in expression of CXCR4, a marker of neutrophil aging. The results demonstrated that the expression of CXCR4 was upregulated over time in medium‐cultured neutrophils, and most neutrophils (>90%) highly expressed CXCR4 after 24 h of stimulation (**Figure** **3**A). Similar observations were made for TTCS‐ and NTCS‐conditioned neutrophils (Figure S5A,B, Supporting Information), and the expression of CXCR4 at 24 and 48 h after stimulation was comparable between medium‐cultured, TTCS‐conditioned, and NTCS‐conditioned neutrophils (Figure S5C, Supporting Information). Subsequently, we quantified the percentage of viable (Annexin^−^PI^−^) CXCR4^+^ neutrophils among the total neutrophils under different stimulation conditions and found that TTCS significantly increased the proportion of viable CXCR4^+^ neutrophils. Notably, blockade of G‐CSF and/or GM‐CSF in TTCS efficiently inhibited the accumulation of viable CXCR4^+^ neutrophils (Figure 3B and Figure S5D, Supporting Information). Provision of exogenous G‐CSF and/or GM‐CSF in NTCS significantly increased the percentage of viable CXCR4^+^ neutrophils (Figure 3B and Figure S5D, Supporting Information). Collectively, these data suggest that delayed neutrophil apoptosis induced by tumor‐derived G‐CSF/GM‐CSF is associated with an increased percentage of viable CXCR4^+^ neutrophils in vitro.

Given the extended lifespan of LSCC‐derived neutrophils, we investigated whether aged CXCR4^+^ neutrophils accumulate in LSCC tissues. Aged neutrophils are characterized by nuclear hypersegmentation and reduced side scatter properties.^[^ ^31^ ^]^ The number of hypersegmented neutrophils increased in the LSCC tissue (Figure 3C). Similarly, the forward and side scatter of LSCC‐derived neutrophils was significantly lower than that of ANT‐derived neutrophils (Figure 3D,E). We then analyzed the proportion of aged neutrophils (CD45^+^CD66b^+^CXCR4^+^CD62L^−^) using flow cytometry. The results indicated that the expressions of CXCR2 and CD62L were decreased in tissue‐derived neutrophils when compared with PB‐derived neutrophils, while the expression of CXCR4 was increased (Figure 3F–H), and LSCC tissues had a higher proportion of aged CXCR4^+^CD62L^−^ neutrophils than ANT (Figure 3I). In addition, we found that the percentage of aged CXCR4^+^ neutrophils was positively correlated with the concentrations of G‐CSF and GM‐CSF, as well as the proportion of viable (Annexin V^−^) neutrophils (Figure 3J–L). Taken together, these findings suggest that delayed neutrophil apoptosis induced by tumor‐derived G‐CSF/GM‐CSF significantly contributes to the accumulation of aged CXCR4^+^ neutrophils in vivo.

### 2.4 Aged CXCR4^+^ Neutrophils Are a Highly Immunosuppressive Subset of Neutrophils in LSCC

**Figure 4:**
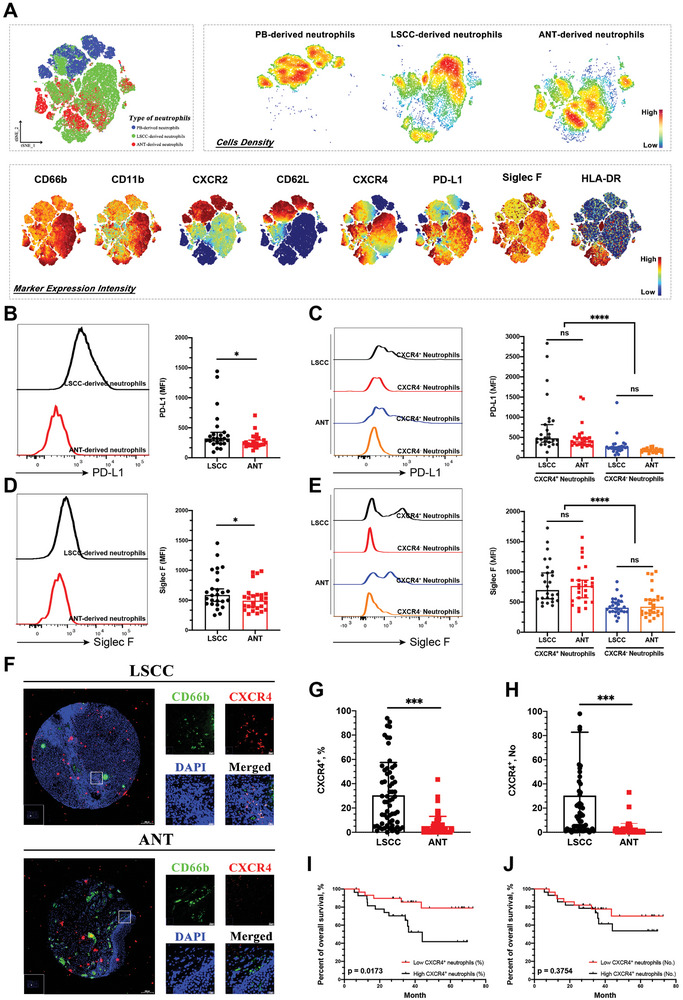
CXCR4^+^ aged neutrophils are highly immunosuppressive and associated with an unfavorable prognosis in LSCC. A) Unsupervised analysis of flow cytometry data gating on CD66b^+^CD45^+^ neutrophils using t‐SNE algorithm (*n* = 15). Density t‐SNE plots of neutrophils derived from PB, ANT, and LSCC and t‐SNE plots of neutrophils overlaid with the marker expression (lower density, blue; higher density, red). B) Representative images of PD‐L1 staining for CD66b^+^CD45^+^ neutrophils (*n* = 26). C) The MFI of PD‐L1 was compared between neutrophils derived from LSCC and ANT, or between groups classified by the positive or negative CXCR4 expression. D) Representative images of Siglec F (CD170) staining for CD66b^+^CD45^+^ neutrophils (*n* = 26). E) The MFI of Siglec F was compared between neutrophils derived from LSCC and ANT, or between groups classified by the positive or negative CXCR4 expression. F) Immunofluorescence staining of CXCR4^+^CD66b^+^ aged neutrophils in LSCC tissues and ANT. Scale bars: 20 µm. G) The proportion of CXCR4^+^ neutrophils among total neutrophils or H) the total count of neutrophils per spot was compared between LSCC and ANT (*n* = 61). I,J) Kaplan‐Meier curves for overall survival stratified by the median percentage of CXCR4^+^ neutrophils (>20%) or the median number of CXCR4^+^ neutrophils (10 per spot). Statistical analysis was conducted using one‐way ANOVA, Mann‐Whitney U tests, and Student's t‐test (****p < 0.0001, ***p < 0.001, **p < 0.01, *p < 0.05). ns, not significant; ANT, adjacent normal tissue; LSCC, laryngeal squamous cell carcinoma; PB, peripheral blood; MFI, mean fluorescence intensity.

Next, we assessed the functional characteristics of aged CXCR4^+^ neutrophils to investigate their role in supporting tumor progression. Flow cytometry was used to determine neutrophil function by focusing on the expression of markers associated with immunosuppression, such as PD‐L1,^[^ ^18^ ^]^ PD‐L2,^[^ ^32^ ^]^ and Siglec F.^[^ ^23^ ^]^ Based on flow cytometry, the t‐SNE algorithm was applied to autologous peripheral blood (PB) and non‐tumor and tumor tissues from 15 patients with LSCC. The results revealed that LSCC‐derived neutrophils had a lower density of CD66b^+^CD11b^+^ cells expressing CXCR2 and CD62L, while displaying an increased density of CD66b^+^CD11b^+^ cells expressing CXCR4, PD‐L1, and Siglec F at baseline than PB‐derived and ANT‐derived neutrophils (**Figure** **4**A). CXCR4^+^ neutrophils demonstrated a higher density of cells expressing PD‐L1 and Siglec F than CXCR4^−^ neutrophils (Figure 4A). We also tested the expression of HLA‐DR in neutrophils, which has been reported to have anti‐tumor antigen‐presenting potency.^[^ ^33^ ^]^ The results showed that the proportion of HLA‐DR^+^ neutrophils was low (approximately 5%) in LSCC, and no significant difference in HLA‐DR expression was observed between CXCR4^+^ and CXCR4^−^ neutrophils.

Subsequently, a broader group of patients with LSCC was included in the supervised analysis of flow cytometry data to confirm and authenticate the initial discoveries. The results showed that LSCC‐derived neutrophils exhibited higher expression of PD‐L1 and Siglec F than ANT‐derived neutrophils (Figure 4B,D). Subgroup analysis indicated that CXCR4^+^ neutrophils derived from both LSCC and ANT expressed significantly higher levels of PD‐L1 and Siglec F than the corresponding CXCR4^−^ neutrophils (Figure 4C,E). Similar trends were observed when analyzing the expression of PD‐L2 and VISTA (Figure S6A–D, Supporting Information) and the secretion levels of ROS, Arg1, and VEGF (Figure S6E–J, Supporting Information). Correlation analyses indicated that the expression of CXCR4 in LSCC‐derived neutrophils positively correlated with the expression of immunosuppressive factors, including PD‐L1, PD‐L2, Siglec F, VISTA, ROS, and Arg1 (Figure S6K, Supporting Information).

Consistent with the flow cytometry findings, double immunofluorescence staining of CD66b and CXCR4 showed that LSCC tissue had a higher infiltration of CXCR4^+^ neutrophils than ANT (Figure 4F–H). Higher infiltration of CXCR4^+^ neutrophils in LSCC was correlated with smoking history and advanced tumor stage (Figure S7, Supporting Information) and was associated with worse survival outcomes (Figure 4I,J and Table S4, Supporting Information). Taken together, these findings demonstrate that aged CXCR4^+^ neutrophils represent a highly immunosuppressive subset within the immune microenvironment of LSCC, potentially contributing to immunosuppression and predicting unfavorable survival.

### 2.5 Neutrophils with Delayed Apoptosis Inhibit the Proliferation and Function of CD8^+^ T Cells

**Figure 5:**
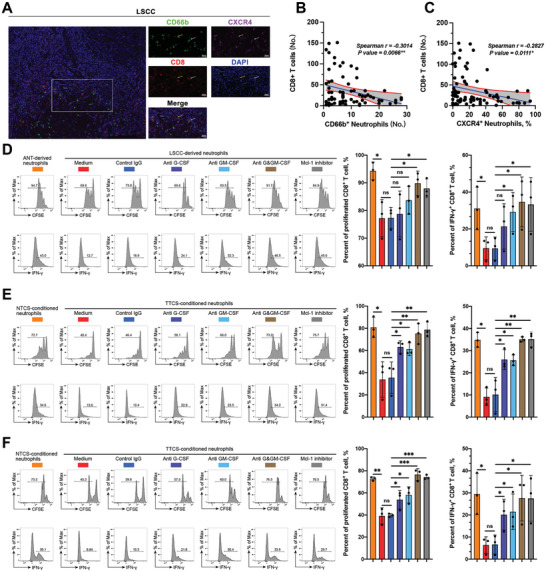
Delayed neutrophil apoptosis contributes to the suppression on CD8^+^ T cell proliferation and IFNγ production by neutrophils. A) Representative images of immunofluorescence staining depicting the colocalization of CD8^+^ T cells and neutrophils (yellow arrow), as well as CXCR4^+^ neutrophils (white arrow) within LSCC. Scale bars: 50 µm. B) Correlation analysis on the presence of CD66b^+^ neutrophils and CD8^+^ T cells within LSCC (*n* = 61). C) Correlation analysis on the presence of CXCR4^+^ neutrophils and CD8^+^ T cells within LSCC (*n* = 61). D) Peripheral CD8^+^ T cells from LSCC patients were labeled using CFSE and subsequently cocultured with autologous LSCC‐derived or ANT‐derived neutrophils for 72 h, which were pretreated with G‐CSF and/or GM‐CSF neutralizing antibodies, antibody against Mcl‐1, or control IgG for 12 h. Representative images and quantification of T cell proliferation and IFNγ‐positive T cells were displayed in a sample size of three. E) Peripheral CD8^+^ lymphocytes from health donors were labeled with CFSE and then cocultured for 72 h with autologous neutrophils, which were pre‐cultured in NTCS or TTCS with or without G‐CSF and/or GM‐CSF neutralizing antibodies, antibody against Mcl‐1, or control IgG for 12 h. Representative images and quantification of T cell proliferation and IFNγ‐positive T cells were shown in a sample size of three. F) Tumor‐specific CD8^+^ T cells were labeled with CFSE and then cocultured for 72 h with autologous neutrophils, which were pre‐cultured in NTCS or TTCS with or without G‐CSF and/or GM‐CSF neutralizing antibodies, antibody against Mcl‐1, or control IgG for 12 h. Representative images and quantification of T cell proliferation and IFNγ‐positive T cells were displayed in a sample size of three. Statistical analysis was conducted using one‐way ANOVA, Mann‐Whitney U tests, and Student's t‐test (***p < 0.001, **p < 0.01, *p < 0.05). ns, not significant; ANT, adjacent normal tissue; LSCC, laryngeal squamous cell carcinoma.

The co‐localization of CD8^+^ T cells and neutrophils, as well as CXCR4^+^ neutrophils, was observed in LSCC tissues in triple immunofluorescence staining of CD8, CD66b, and CXCR4 (**Figure** **5**A). Correlation analysis indicated that the infiltration of CD8^+^ T cells negatively correlated with the infiltration of neutrophils and CXCR4^+^ neutrophils (Figure 5B,C). These findings suggest that the presence of neutrophils, particularly CXCR4^+^ neutrophils, hinders the immune response of CD8^+^ T cells and potentially facilitates tumor progression. To investigate this further, we isolated neutrophils from non‐tumor and tumor samples from patients with autologous LSCC. After pretreatment, as mentioned above, these neutrophils were co‐cultured with purified PB CD8^+^ T cells from the same individuals for 3 d. The results demonstrated that LSCC‐derived neutrophils exhibited greater effectiveness in suppressing the proliferation and IFNγ secretion of CD8^+^ T cells compared to ANT‐derived neutrophils. This effect was effectively diminished by inhibiting the Mcl‐1, G‐CSF, and/or GM‐CSF signaling pathways in neutrophils (Figure 5D). These data suggest an immunosuppressive function of life‐prolonged tumor‐infiltrating neutrophils in tumor immunity that can be effectively counteracted by promoting neutrophil apoptosis.

Given that LSCC‐derived neutrophils exhibited a greater inhibitory effect on CD8^+^ T lymphocytes than ANT‐derived neutrophils, it is plausible that the LSCC microenvironment may be crucially involved in facilitating this process. To test this hypothesis, we co‐cultured purified PB CD8^+^ T cells with NTCS‐ or TTCS‐conditioned autologous PB neutrophils for 3 d. Notably, the proliferation and IFNγ secretion of CD8^+^ T cells were significantly suppressed by TTCS‐conditioned neutrophils, and this suppressive effect could be reduced by blocking G‐CSF and/or GM‐CSF or using an anti‐Mcl‐1 antibody on neutrophils (Figure 5E).

To provide a more direct demonstration of the suppressive function exerted by neutrophils, tumor‐specific CD8^+^ T cells were generated and co‐cultured with autologous purified PB neutrophils conditioned with either NTCS or TTCS. We found that TTCS‐conditioned neutrophils exhibited a notable ability to inhibit the proliferation and IFNγ production of tumor‐specific CD8^+^ T cells, which occurred in a delayed, apoptosis‐dependent manner (Figure 5F). These results indicate that delayed neutrophil apoptosis promotes the ability of neutrophils to suppress CD8^+^ T cell function in the LSCC microenvironment.

### 2.6 Neutrophils with Delayed Apoptosis Accelerate the Exhaustion of CD8^+^ T Cells

**Figure 6:**
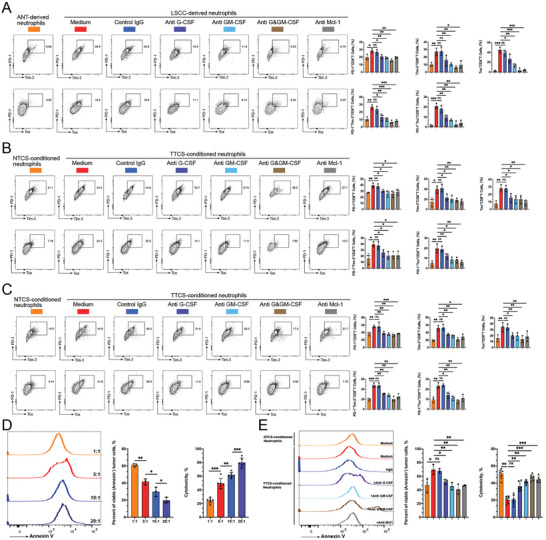
Prolonged neutrophil survival contributes to the acceleration of CD8^+^ T cell exhaustion by neutrophils. A) Peripheral CD8^+^ T cells from LSCC patients were cocultured for 72 h with autologous LSCC‐derived or ANT‐derived neutrophils that were pretreated with G‐CSF and/or GM‐CSF neutralizing antibodies, antibody against Mcl‐1, or control IgG for 12 h. Representative images and quantification of PD‐1^hi^Tim‐3^+^ or PD‐1^hi^Tox^+^ terminally exhausted T cells are displayed in a sample size of three. B) PB‐derived CD8^+^ T lymphocytes from healthy donors were cocultured for 72 h with autologous neutrophils that were precultured in NTCS or TTCS with or without G‐CSF and/or GM‐CSF neutralizing antibodies, antibody against Mcl‐1, or control IgG for 12 h. Representative images and quantification of PD‐1^hi^Tim‐3^+^ or PD‐1^hi^Tox^+^ terminally exhausted T cells are shown in a sample size of three. C) Tumor‐specific CD8^+^ T lymphocytes were cocultured for 72 h with autologous neutrophils that were precultured in NTCS or TTCS with or without G‐CSF and/or GM‐CSF neutralizing antibodies, antibody against Mcl‐1, or control IgG for 12 h. Representative images and quantification of PD‐1^hi^Tim‐3^+^ or PD‐1^hi^Tox^+^ terminally exhausted T cells are displayed in a sample size of three. D) Tumor‐specific CD8^+^ T lymphocytes were co‐cultured with tumor cells (Tu686) at various E:T ratios for 48 h. The cytotoxicity of tumor‐specific CD8^+^ T cells on tumor cells were analyzed using Annexin V/PI staining and LDH assay. F) Tumor‐specific CD8^+^ T lymphocytes were cocultured for 72 h with neutrophils that were pre‐incubated in NTCS or TTCS with or without the presence of G‐CSF and/or GM‐CSF neutralizing antibodies, antibody against Mcl‐1, or control IgG for 12 h. Tumor‐specific CD8^+^ T cells were then harvested and cultured with Tu686 cells for 48 h. The cytotoxicity of tumor‐specific CD8^+^ T cells on Tu686 cells was analyzed using Annexin V/PI staining and LDH assay (*n* = 3). Statistical analysis was conducted using one‐way ANOVA, Mann‐Whitney U tests, and Student's t‐test (***p < 0.001, **p < 0.01, *p < 0.05). ns, not significant.

Recent studies have reported CD8^+^ T cell exhaustion in cancer.^[^ ^34^ ^]^ We sought to delineate the role of apoptosis‐delayed neutrophils in the progression of CD8^+^ T cell exhaustion. We cocultured purified PB CD8^+^ T cells with autologous LSCC‐ or ANT‐derived neutrophils for 3 d. Compared with ANT‐derived neutrophils, LSCC‐derived neutrophils significantly upregulated the frequency of PD‐1^hi^Tim‐3^+^ as well as PD‐1^hi^Tox^+^ “terminally exhausted” CD8^+^ T cell populations, and this upregulation could be efficiently attenuated by blocking Mcl‐1 or G‐CSF and/or GM‐CSF on neutrophils (**Figure** **6**A). A similar increase of PD‐1^hi^Tim‐3^+^ and PD‐1^hi^Tox^+^ “terminally exhausted” CD8^+^ T cells were observed in the co‐culture system of purified PB CD8^+^ T cells and NTCS/TTCS‐conditioned neutrophils (Figure 6B), as well as tumor‐specific CD8^+^ T cells and NTCS/TTCS‐conditioned neutrophils (Figure 6C). Moreover, we assessed the relationship among delayed neutrophil apoptosis, CXCR4^+^ neutrophils, and exhausted CD8^+^ T cells. The results showed that the proportion of viable (Annexin V^−^) neutrophils was significantly associated with the proportion of PD‐1^+^CD8^+^ T cells. Positive correlations were also observed between viable neutrophils and Tim‐3^+^CD8^+^ T cells or Tox^+^CD8^+^ T cells (Figure S8, Supporting Information), although the p values were not statistically significant. In terms of immunosuppressive CXCR4^+^ neutrophils, which accumulate in LSSC tissues and are correlated with delayed apoptosis, similar results were observed for their relationship with exhausted CD8^+^ T cells. These data collectively demonstrate that within the LSCC microenvironment, delayed apoptosis of neutrophils promotes their acquisition of the ability to accelerate the terminal exhaustion of CD8^+^ T cells.

### 2.7 Targeting Delayed Apoptosis of Neutrophils Restores the Cytotoxicity of Tumor‐Specific CD8^+^ T Cells on Tumor Cells In Vitro and In Vivo

Since neutrophils with delayed apoptosis can impair the function and accelerate the exhaustion of tumor‐specific CD8^+^ T cells, it is worth investigating whether they also have a substantial impact on the cytotoxicity of tumor‐specific CD8^+^ T cells on tumor cells. To test this possibility, we first incubated tumor‐specific CD8^+^ T cells with tumor cells at various E:T ratios for 48 h and tested the tumor‐killing ability of tumor‐specific CD8^+^ T cells using Annexin V/PI staining and lactate dehydrogenase (LDH) assay. The data showed that tumor cells were effectively eliminated by tumor‐specific CD8^+^ T cells in a ratio‐dependent manner (Figure 6D). Tumor‐specific CD8^+^ T cells were then co‐cultured with NTCS‐ or TTCS‐conditioned neutrophils. These neutrophils had been pre‐treated with G‐CSF and/or GM‐CSF‐neutralizing antibodies or anti‐Mcl‐1 antibody for 72 h, as mentioned previously. Next, T cells were harvested and incubated with tumor cells for 72 h. The findings indicated that TTCS‐conditioned neutrophils significantly inhibited the cytotoxicity of tumor‐specific CD8^+^ T cells, and this effect could be effectively attenuated by blocking G‐CSF and/or GM‐CSF or using anti‐Mcl‐1 antibody on neutrophils (Figure 6E).

**Figure 7:**
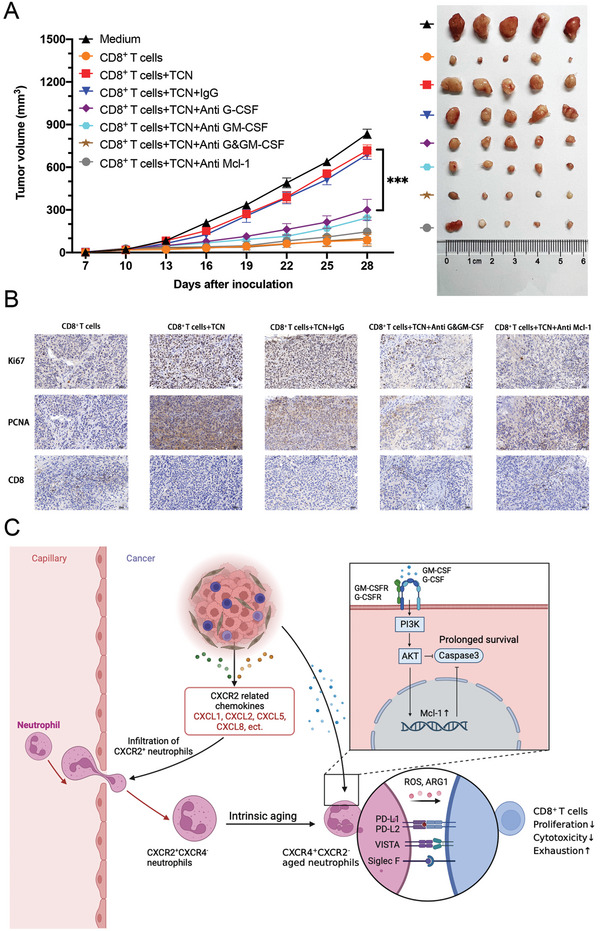
Delayed neutrophil apoptosis contributes to the neutrophils’ suppression on CD8^+^ T cells’ cytotoxicity and promotion of tumor growth in vivo. A) Tumor‐specific CD8^+^ T cells were first cocultured for 72 h with autologous neutrophils, which were pre‐cultured in TTCS with or without G‐CSF and/or GM‐CSF neutralizing antibodies, antibody against Mcl‐1, or control IgG for 12 h. On the 10th day after tumor inoculation, tumor‐bearing mice received an injection of buffered saline (medium) or tumor‐specific CD8^+^ T cells, which were prepared as described above. The volumes of the tumors were documented from the day of injection of Tu686 cells (designated as day 0) and analyzed for inter‐group differences. Excision and photography of the tumors were conducted on day 28 after the injection of Tu686 cells. B) Representative images of immunohistochemical staining showing the expressions of PCNA and Ki67 in tumors. Scale bars: 50 µm. C) A proposed framework elucidating the interplay between neutrophils, CD8^+^ T cells, and tumor cells resulting in immunosuppression mediated by lifespan‐extended neutrophils and subsequent tumor progression in LSCC. ***p < 0.001. TCN, TTCS‐conditioned neutrophils; PCNA, proliferating cell nuclear antigen.

To validate the above findings in vivo, PB‐derived neutrophils were treated with TTCS with or without G‐CSF and/or GM‐CSF‐neutralizing antibody or anti‐Mcl‐1 antibody and then co‐cultured with autologous tumor‐specific CD8^+^ T lymphocytes for 72 h. Tumor‐specific CD8^+^ T lymphocytes were intraperitoneally injected into non‐obese diabetic/severe combined immunodeficient (NOD/SCID) mice bearing human Tu686‐derived LSCC. As anticipated, tumor‐bearing mice lacking T cell transplantation or mice receiving T cells precultured with TTCS‐conditioned neutrophils (TCNs) or IgG‐treated TCNs showed disease progression and tumor growth (**Figure** **7**A). Mice receiving T cells pre‐cultured with G‐CSF and/or GM‐CSF or Mcl‐1 antibody‐treated TCNs exhibited decreased tumor progression (Figure 7A), demonstrating the critical role of neutrophils with delayed apoptosis in assisting tumor growth in vivo. In addition, mice receiving injections of T cells that were pre‐cultured with G‐CSF/GM‐CSF or Mcl‐1 antibody‐treated TCNs exhibited increased CD8^+^ T cell infiltration and decreased Ki67‐ or PCNA‐positive cells, indicating reduced proliferation of tumor cells (Figure 7B). Collectively, these findings indicate that TANs with delayed apoptosis could assist in LSCC progression by suppressing tumor‐specific CD8^+^ T cell immunity.

## 3 Discussion

The role of neutrophils in promoting tumor growth is gaining attention; however, our knowledge of their adaptability and reaction to diseases remains insufficient. In this study, we investigated the delayed apoptosis of neutrophils that infiltrate LSCC tissues. We utilized various complementary approaches to uncover the mechanisms responsible for inducing this delay as well as to explore the phenotype, biological functions, and clinical significance of neutrophils with delayed apoptosis in the TME of patients with LSCC. Our findings indicated that LSCC‐infiltrating neutrophils with delayed apoptosis play an active role in immune suppression and tumor progression. To our knowledge, this is the first demonstration of tumor‐derived G‐CSF and GM‐CSF collectively inducing delayed neutrophil apoptosis and the accumulation of immunosuppressive aged neutrophils in LSCC, which exhibits a significant correlation with adverse patient prognosis. Furthermore, this study provides novel insights into the inhibitory effects of these apoptosis‐delayed neutrophils on tumor‐specific CD8^+^ T cell immunity, thereby promoting tumor progression.

Neutrophil infiltration in LSCC tumors has been previously documented,^[^ ^35^, ^36^ ^]^ and its correlation with disease progression was confirmed in our study using collected LSCC samples. In addition, based on the difference in neutrophil infiltration between early‐ and late‐stage patients measured by immunohistochemistry or flow cytometry, we speculate that advanced tumors could enhance neutrophil infiltration via the secretion of chemokines (e.g., CXCL5)^[^ ^37^ ^]^ because late‐stage patients have higher numbers of circulating neutrophils. This may also be due to the increased accumulation of long‐lived neutrophils within the tumor, suggesting the need to explore the mechanism of delayed neutrophil apoptosis. However, the precise mechanisms underlying the regulation of neutrophil apoptosis and lifespan in patients with LSCC are not fully understood. Previous studies have indicated that inflammatory cytokines like IL‐1β and G‐CSF may potentially extend neutrophil survival.^[^ ^38^, ^39^ ^]^ Here, we identified G‐CSF and GM‐CSF derived from LSCC cells as novel proinflammatory factors that can effectively delay neutrophil apoptosis via the activation of the PI3K‐AKT signaling pathway. Murphy and Caraher described the role of anti‐apoptotic Bcl‐2 family proteins in neutrophil apoptosis and addressed their importance in neutrophil homeostasis.^[^ ^40^ ^]^ Consistent with their findings, our data showed that the activation of the PI3K‐AKT signaling pathway has the potential to increase the levels of the anti‐apoptotic protein Mcl‐1, which then abrogates the cleavage and activation of procaspase‐3. A recent study by Bodac et al.^[^ ^41^ ^]^ reported that tumor cell‐derived GM‐CSF triggered the expression of the anti‐apoptotic Bcl‐xL protein and enhanced neutrophil survival in a genetically engineered mouse model of lung adenocarcinoma, which differed slightly from our human cell‐based study. The mechanistic differences by which GM‐CSF regulates neutrophil survival between studies are interesting, and these differences might be caused by cancer type or species discrepancies, which requires further validation and investigation. In addition to apoptosis, previous studies have reported the involvement of other neutrophil death pathways, including eryptosis, necroptosis, pyroptosis, and NETosis.^[^ ^42^ ^]^ The multifaceted aspects of neutrophil death induced by different factors may also play a role in prolonging the lifespan of neutrophils. However, this requires further investigation. Circulating apoptotic neutrophils usually return to the BM and are cleared by macrophages. The mechanisms by which tumor‐infiltrating neutrophils resist the efferocytosis of macrophages in the TME of LSCC also warrant further exploration.

Neutrophils released from BM exhibit elevated levels of CD62L, which gradually diminish throughout the day. Simultaneously, there is an augmentation of surface CXCR4, which is a phenomenon commonly referred to as aging. Our data demonstrate that similar to circulating neutrophils, tumor‐infiltrating neutrophils also undergo an aging process characterized by the upregulation of CXCR4 expression. The expression of CXCR4 in tumor‐conditioned neutrophils was synchronized with that of medium‐conditioned neutrophils and remained unaffected by extrinsic factors, which aligns with previous findings indicating that neutrophil aging is an intrinsic circadian process.^[^ ^29^ ^]^

Interestingly, our data showed that the extended lifespan of neutrophils could greatly contribute to the increased percentage and accumulation of aged CXCR4^+^ neutrophils within the tumor. Accumulated aged neutrophils have been reported to accelerate vascular damage and induce tumor progression by promoting lung metastasis in breast cancer through the formation of vital mitochondria‐dependent NETs.^[^ ^43^ ^]^ Here, we observed that CXCR4^+^ neutrophils within tumors expressed significantly higher levels of PD‐L1 and Siglec F than the corresponding CXCR4^−^ neutrophils. Comparable findings were also noted regarding the expression levels of PD‐L2 and VISTA as well as the secretion of ROS and Arg1. Previous studies have reported immunosuppressive functions of neutrophils expressing these molecules.^[^ ^44^, ^45^ ^]^ Tang et al. reported that tumor‐infiltrating PD‐L1^+^ neutrophils facilitate T cell immunity and promote tumor progression.^[^ ^18^ ^]^ Therefore, we propose that LSCC‐infiltrating CXCR4^+^ neutrophils, which accumulate within the tumor, are immunosuppressive and protumorigenic. Gao et al. identified HLA‐DR^+^CD74^+^ neutrophils with anti‐tumor antigen‐presenting potency that showed cancer‐type preferences. They reported the decreased infiltration of HLA‐DR^+^CD74^+^ neutrophils in oral squamous cell carcinoma (OSCC), which was consistent with our findings. Therapies targeting immunosuppressive CXCR4^+^ neutrophils and activating HLA‐DR^+^CD74^+^ neutrophils may have synergistic effects in LSCC treatment.

T cells, particularly CD8^+^ T cells, predominantly suppress tumor growth.^[^ ^46^, ^47^ ^]^ Previous studies have reported that neutrophils exhibit high plasticity and remarkable adaptive abilities in response to different TME cues.^[^ ^48^ ^]^ Tumor‐infiltrating neutrophils can acquire a T cell suppressor phenotype characterized by inhibition of T cell activation and proliferation.^[^ ^49^ ^]^ Consistent with these findings, we observed that both tumor‐infiltrating neutrophils and TTCS‐conditioned circulating neutrophils showed suppressive effects on activated CD8^+^ T cell proliferation and IFN‐𝛾 secretion. Notably, no study has investigated the role of delayed neutrophil apoptosis on T cell immunity within human malignancy. Here, we observed a notable decrease in the inhibitory effect of tumor‐infiltrating and TTCS‐conditioned neutrophils on the proliferation of CD8^+^ T cells and their production of IFN‐γ after the reversal of delayed apoptosis. Notably, recent studies reported that various factors derived from the TME could induce the exhaustion and dysfunction of T cells.^[^ ^50^, ^51^ ^]^ The phenotype of exhausted T cells is characterized by decreased cytokine production, gradual loss of robust cytotoxicity, transcriptional alteration (e.g., Foxo1, Tox, and Nfatc1), and upregulation of various inhibitory receptors (e.g., PD‐1, Tim‐3, CTLA4, and BTLA).^[^ ^51^, ^52^, ^53^ ^]^ How do TANs act on the phenotypic transformation of exhausted T cells? Here, we are the first to elucidate that both tumor‐infiltrating neutrophils and TTCS‐conditioned circulating neutrophils could promote the exhaustion of CD8^+^ T cells. Additionally, the reversal of delayed apoptosis in neutrophils resulted in a significant deceleration of CD8^+^ T cell exhaustion within the co‐culture system comprising neutrophils and CD8^+^ T cells.

The molecular mechanisms by which suppressor neutrophils exacerbate the dysfunction and exhaustion of CD8^+^ T cells are complex and require further investigation. In the present study, delayed neutrophil apoptosis appeared to promote the accumulation of suppressive CXCR4^+^ neutrophils and the immunoparalysis of CD8^+^ T cells. Considering the abnormally prolonged lifespan of neutrophils in the TME, we hypothesize that reversing delayed apoptosis in tumor‐infiltrating neutrophils could partially restore CD8^+^ T cell immunity, irrespective of the suppressive mechanisms underlying the interaction between neutrophils and CD8^+^ T cells. Based on these findings, we propose that targeting the prolonged lifespan of neutrophils may be effective in converting the immunosuppressive niche of the TME into a more active one.

In conclusion, our findings elucidate a novel mechanism whereby tumor‐derived G‐CSF and GM‐CSF induce delayed neutrophil apoptosis by activating the PI3K‐AKT signaling pathway. Moreover, delayed neutrophil apoptosis can facilitate the accumulation of immunosuppressive CXCR4^+^ neutrophils and impair CD8^+^ T cell immunity. The findings of our study contribute an additional perspective to the understanding of the immunosuppressive mechanisms of tumor‐infiltrating neutrophils. In the future, therapeutic interventions targeting the prolonged lifespan of neutrophils under pathological conditions could potentially emerge as a viable approach for enhancing anti‐tumor immune responses.

## 4 Experimental Section

### Study Participants and Specimens

Samples were collected from patients with LSCC who underwent curative resection at the Department of Otolaryngology, Eye, Ear, Nose and Throat Hospital, Fudan University. Patients with a history of multiple primary malignancies or previous chemotherapy or radiation treatment were excluded from the study. The first cohort comprised 61 patients with LSCC who underwent surgical resection between June 2014 and September 2017. Fresh tumor tissues (without necrotic foci) and adjacent normal tissues (ANT; located >5 cm from the LSCC site) were used for tumor microarray immunohistochemistry and immunofluorescence analysis. Detailed clinicopathological data for this cohort are presented in Table S1 (Supporting Information). For Cohort 2, fresh LSCC tissues, ANT, and autologous PB were collected from patients with LSCC between September 2021 and July 2023 (detailed clinicopathological information is provided in Table S1, Supporting Information). Peripheral blood samples from age‐ and sex‐matched healthy donors were used as controls. These tissue and blood samples were used to perform flow cytometric analysis and assess their functional attributes. The antibodies and reagents used in this study are listed in Table S2 (Supporting Information).

This study was conducted following the principles outlined in the Declaration of Helsinki and was approved by the Ethics Committee of the Eye, Ear, Nose and Throat Hospital, Fudan University, Shanghai, China (No. KJ2008‐01). Written informed consent was obtained from all patients.

### Immunohistochemistry

Paraformaldehyde‐fixed and paraffin‐embedded samples from the first cohort were processed for tissue microarray (TMA) construction according to previously described methods.^[^ ^54^ ^]^ The TMA slides were subjected to deparaffinization, rehydration, antigen retrieval, and blocking. Subsequently, each slide was incubated with mouse anti‐human CD66b overnight at 4 °C in a humid chamber and then stained with biotin‐conjugated secondary anti‐mouse/rabbit IgG for 1 h at room temperature. Samples obtained from animal experiments were fixed with paraformaldehyde and embedded in paraffin. The samples were then sectioned into 4 µm thick slices and placed on slides. The slides were incubated with anti‐human proliferating cell nuclear antigen (PCNA) and anti‐human Ki67 antibodies separately, followed by incubation with biotin‐conjugated secondary anti‐mouse/rabbit IgG. Cells with brown membranous staining were counted, either the total spot or three randomly selected high‐power fields (400×) per sample, to quantify positive labeling.

### Immunofluorescence

TMA slides were incubated overnight at 4 °C with mouse anti‐CD66b antibody and rabbit anti‐CXCR4 or rabbit anti‐CD8 antibody. After three washes with phosphate‐buffered saline (PBS) for 10 min each, the slides were incubated in the dark with secondary antibodies conjugated to different fluorochromes for 60 min. After washing, all slides were counterstained with 4′,6‐diamidino‐2‐phenylindole (DAPI) and mounted using Vectashield HardSet Mounting Medium.

### Preparation of Single‐Cell Suspension

After three washes with PBS supplemented with 5% penicillin/streptomycin (Invitrogen), fresh tissues were finely minced and incubated in complete RPMI‐1640 medium supplemented with DNase I (0.25 mg mL^−1^, Sigma‐Aldrich), collagenase IV (1 mg mL^−1^, Sigma‐Aldrich), and 5% fetal bovine serum (FBS; HyClone) at 37 °C for 45 min. The digested tissues were subsequently passed through a 70 µm strainer (Corning) and centrifuged at 1500 rpm for 5 min. After resuspension, the erythrocytes were lysed using Red Blood Cell Lysis Buffer (Yeasen Biotechnology, Shanghai, China). Lymphocyte Separation Medium (Yeasen Biotechnology) was used for density gradient centrifugation to isolate peripheral blood mononuclear cells (PBMCs) from patients with LSCC or healthy donors. Neutrophils were obtained from the blood samples after the elimination of erythrocytes.

### Neutrophil Isolation and Cell Morphology

Using single‐cell suspensions of fresh tissue, tissue‐infiltrating neutrophils were isolated using the Easysep CD66b positive selection kit (StemCell) according to the manufacturer's instructions. For neutrophil morphology staining, the neutrophils were smeared onto glass slides and fixed with 4% paraformaldehyde (Yeasen Biotechnology) before Giemsa staining.

### Preparation of Tissue or Cell Culture Supernatants

Fresh non‐tumor or tumor tissues were rinsed three times with PBS supplemented with 5% penicillin/streptomycin (Invitrogen). Tumor tissue culture supernatants (TTCS) or non‐tumor tissue culture supernatants (NTCS) were obtained by incubating 50 mg fresh tumor or non‐tumor tissue in 1 mL of complete RPMI‐1640 medium for 24 h. After centrifugation, the supernatants were collected.

To prepare the tumor cell culture supernatant (TCCS) or non‐tumor cell culture supernatant (non‐TCCS), single cells of fresh non‐tumor or tumor tissues were harvested as described above and washed three times with PBS. Subsequently, the cells were suspended in complete F medium, which was a mixture of complete DMEM (DMEM supplemented with 1% L‐glutamine, 1% penicillin/streptomycin, and 10% FBS) and F‐12 nutrient mix (3:1; v/v). The complete F medium was further supplemented with ROCK inhibitor Y‐27632 (10 × 10^−6^ m; Selleck), amphotericin B (250 ng mL^−1^; Millipore Sigma), insulin (5 µg mL^−1^; Millipore Sigma), hydrocortisone (25 ng mL^−1^; Sigma‐Aldrich), and epidermal growth factor (0.125 ng/mL; Thermo Fisher Scientific). After incubation for 48 h, the cells adhered to the dish, and the medium was replaced every 3 d. Cancer‐associated fibroblasts (CAFs) were selectively removed by differential trypsinization using 0.25% trypsin‐EDTA at room temperature for 2 min. For digestion of cancer cells, 0.25% trypsin–EDTA was used at 37 °C for 7 min. The purity of the cancer cells was assessed by flow cytometric analysis based on Ckpan expression levels, ensuring that only samples containing >90% Ckpan^+^ cells were used in subsequent experiments. TCCS and non‐TCCS were obtained by plating either 5 × 10^6^ tumor cells or non‐tumor cells in 1 mL complete RPMI‐1640 medium for 24 h, followed by harvesting by centrifugation.

### Neutrophil Stimulation

To obtain conditioned neutrophils, blood neutrophils were cultured with 50% NTCS (diluted in complete RPMI‐1640 medium), 50% TTCS, 50% TCCS, or 50% non‐TCCS for specific durations. Alternatively, the cells were cultured with different concentrations of TTCS or TCCS (25%, 50%, or 75%) for either 24 or 48 h. Neutrophils were cultured with either 50% TTCS or TCCS in the presence of neutralizing antibodies against GM‐CSF and/or G‐CSF. Furthermore, cells were cultured with either 50% NTCS or 50% non‐TCCS, along with human recombinant GM‐CSF and/or G‐CSF as well as other cytokines, for a period of either 24 or 48 h. Subsequently, neutrophils were harvested and washed for western blot analysis and flow cytometry. For the signaling pathway inhibition experiment, neutrophils were stimulated with 50% TTCS in combination with specific inhibitors targeting PI3K, AKT, NFκB, JNK, JAK, ERK, MEK, STAT3, or mTOR individually. Details of these inhibitors are presented in Table S2 (Supporting Information). Because dimethyl sulfoxide (DMSO) was used as the solvent for all inhibitors, the neutrophil control groups were subjected to culture medium supplemented with DMSO.

### In Vitro Coculture of Neutrophils and Peripheral CD8^+^ T Cells

Three coculture systems were used to investigate the effects of neutrophils with delayed apoptosis on the proliferation and function of CD8^+^ T cells. For the first in vitro coculture system, magnetic bead‐purified peripheral CD8^+^ T cells (2 × 10^5^ cells per well in 96‐well round‐bottom plates) were stained with CFSE (Thermo Fisher Scientific) and co‐cultured with autologous tissue‐infiltrating neutrophils isolated from ANT or LSCC tissues in a 1:1 ratio for 3 d. This coculture was performed in 200 µL complete RPMI‐1640 medium supplemented with anti‐CD28 antibody (1 µg mL^−1^), anti‐CD3 antibody (2 µg mL^−1^), and rhIL2 (20 IU mL^−1^). Before culturing with CD8^+^ T cells, tissue‐infiltrating neutrophils were subjected to a 12 h pretreatment in complete RPMI‐1640 medium with or without neutralizing antibodies against G‐CSF and/or GM‐CSF, anti‐Mcl‐1 antibody, or control IgG. For the second in vitro co‐culture system, CFSE‐labeled magnetic bead‐purified peripheral CD8^+^ T cells (2 × 10^5^ cells per well in 96‐well round‐bottom plates) were cocultured with autologous peripheral neutrophils at a 1:1 ratio, as described above. These neutrophils were subjected to a 12 h pretreatment with either 50% NTCS or TTCS in the presence or absence of neutralizing antibodies against G‐CSF and/or GM‐CSF, anti‐Mcl‐1 antibody, or control IgG.

For the third in vitro coculture system, we investigated how different neutrophils affected the proliferation and activity of tumor‐specific CD8^+^ T cells stimulated by tumor antigens presented by tumor‐loaded dendritic cells (DCs). Briefly, monocyte‐derived DCs from healthy donors were activated by incubation with irradiated apoptotic Tu686 cells at a ratio of 1:6 for 1 d. Autologous peripheral CD8^+^ T cells (2 × 10^5^ cells per well in 96‐well round‐bottom plates) were then incubated with the tumor‐loaded DCs in 200 µL complete RPMI‐1640 medium containing anti‐CD28 antibody (1 µg mL^−1^), anti‐CD3 antibody (2 µg mL^−1^), rhIL2 (20 IU mL^−1^), and rhIL7 (10 ng mL^−1^) at a ratio of 10:1 for 2 weeks. Subsequently, the CD8^+^ T cells were exposed to new tumor‐loaded DCs for an additional 2 week period to cultivate tumor‐specific CD8^+^ T cells. CFSE‐labeled tumor‐specific CD8^+^ T cells were cocultured with autologous TTCS‐ or NTCS‐conditioned peripheral neutrophils, pretreated as described above. After a 3 d incubation period, the cells were collected for flow cytometry.

### In Vitro T Cell Cytotoxicity on Tumor Cells

The cytotoxic effects of tumor‐specific CD8^+^ T cells were assessed using the Annexin V‐FITC/propidium iodide (PI) Apoptosis Detection Kit from Yeasen Biotechnology and the CyQUANTTM lactate dehydrogenase (LDH) Cytotoxicity Assay Kit from Millipore Sigma. Tumor‐specific CD8^+^ T cells were cocultured with autologous NTCS‐ or TTCS‐conditioned peripheral neutrophils, which were pretreated as described above, at a ratio of 2:1 for 72 h. Subsequently, tumor‐specific CD8^+^ T cells were collected and incubated with Tu686 cells in complete RPMI‐1640 medium containing rhIL2 in a 96‐well plate at ratios of 1:1, 3:1, and 10:1 for 48 h. The cells were then collected and stained with anti‐human CD3 (#552852, BD Biosciences) and Annexin V/PI staining solution. Flow cytometry (LSR Fortessa, BD Biosciences) was performed to detect apoptosis in CD3‐negative Tu686 cells.

For the LDH cytotoxicity assay after the co‐culture of tumor‐specific CD8^+^ T cells and Tu686 cells as described above, 50 µL of supernatant per well was collected to measure LDH release at an absorbance of 490 nm using a microplate reader. As a control, maximum LDH activity was determined by adding 1× lysis buffer (Thermo Fisher Scientific) to the Tu686 cell culture system, while spontaneous LDH activity was assessed by incubating Tu686 cells alone. The percentage of cytotoxicity exerted by CD8^+^ T cells specific to the tumor cells was calculated using the following equation: %cytotoxicity = [(experimental LDH activity − baseline LDH activity)/(maximum LDH activity − baseline LDH activity)] × 100%.

### In Vivo Tumor Inhibition Assay

All animal experiments were conducted in compliance with ethical standards and approved by the Institutional Animal Care and Use Committee of Fudan University (No. 202210121S). Male NOD/SCID mice (Gempharmatech, Jiangsu, China) aged between 5 and 7 weeks were used. To establish tumor‐bearing mice, 5 × 10^5^ LSCC cells (Tu686) were subcutaneously injected into the right armpit of each mouse. Tumor‐specific CD8^+^ T cells (5 × 10^6^) were cocultured with autologous peripheral TTCS‐ or NTCS‐conditioned neutrophils, pretreated as mentioned earlier, at a ratio of 2:1 for 72 h. After this incubation period, the tumor‐specific CD8^+^ T cells were collected and suspended in buffered saline solution (100 µL). They were then administered via peritoneal injection 10 d after tumor inoculation. The size and volume of the tumors were measured every 3 d by two independent observers to plot growth curves accordingly. After sacrificing the mice, the tumors were photographed, weighed, fixed in 4% (w/v) paraformaldehyde, and embedded in paraffin. Paraffin sections were immunohistochemically stained.

### Flow Cytometry

Flow cytometry was performed in accordance with standard protocols as previously described.^[^ ^18^ ^]^ For cell surface marker staining, cell suspensions were incubated using a Zombie UV Fixable Viability Kit (423107, BioLegend) and fluorescently labeled antibodies for 20 min in the dark at room temperature. For intracellular and/or intranuclear staining, the cells were first subjected to surface staining and then fixed and permeabilized according to the manufacturer's instructions (#00‐5523‐00, Invitrogen), followed by staining with the indicated antibodies. All antibodies used are listed in Table S2 (Supporting Information). After staining, the cells were washed and then measured using an LSR Fortessa (BD Biosciences), and data analysis was conducted using R software (version 3.5.0, cytofkit package) or FlowJo software (TreeStar). Supervised analysis of flow cytometry data was performed by a single operator who was blinded to the clinical information of the patients. Unsupervised data analysis was performed using the t‐distributed stochastic neighbor embedding (t‐SNE) algorithm. After the compensation matrix setup, CD66b^+^ cell events were extracted, and logicle transformation was applied. t‐SNE analysis was performed on 3000 CD66b^+^ cells for each specimen. In some cases, the cells were directly incubated with Annexin V/PI (Yeasen Biotechnology) prior to flow cytometric analysis.

### Western Blot

Cellular extracts were obtained by isolating intact cells in RIPA lysis buffer supplemented with phosphatase and protease inhibitors (Servicebio). The cell lysate proteins (20 µL) were electrophoresed on 10% SDS‐PAGE gels and transferred to polyvinylidene fluoride (PVDF) membranes. The membranes were blocked with 5% nonfat milk in Tris‐buffered saline Tween‐20 (TBST), and subsequently subjected to overnight incubation with specific primary antibodies at 4 °C. All the primary antibodies were purchased from Cell Signaling Technology (PI3K #4249; pPI3K #4228; AKT #4685; pAKT #4060; NFκB #4810; MCL‐1 #5453; ProCaspase‐3 #9662; Cleaved Caspase 3 #9661, Table S2, Supporting Information). Thereafter, the membranes were washed with TBST and incubated with a secondary goat anti‐mouse/rabbit IgG HRP conjugated antibody (CST). After three washes with TBST, the blots were developed using the enhanced chemiluminescence method (EpiZyme).

### Enzyme‐Linked Immunosorbent Assay (ELISA)

Tissue culture supernatants were collected for ELISA as previously described. The levels of G‐CSF (#EK169HS, MultiSciences) and GM‐CSF (#EK163HS, MultiSciences) were measured according to the manufacturer's instructions.

### Statistical Analysis

The detailed statistical test methods, sample sizes, and *p* values used in this study are indicated in the corresponding legends. GraphPad Prism 9 (version 9.4) was used for all the statistical analyses. All results are presented as the mean ± standard error of the mean (SEM). Student's t‐test was used to compare two groups when the data followed a normal distribution; otherwise, the nonparametric Mann‐Whitney U test was used. Spearman correlation analysis was performed to evaluate the correlations between the quantitative parameters owing to their non‐normal distribution. Survival analyses were performed using the Kaplan‐Meier method, and survival differences between independent groups were compared using the log‐rank test. Hazard ratios (HRs) and the corresponding 95% confidence intervals (CIs) were calculated using univariate or multivariate Cox proportional hazards models. All statistical tests were two‐tailed, and a *p* value <.05 indicated statistical significance.

### Ethics Approval Statement

This study adhered to the principles of the Declaration of Helsinki and received approval from the Medical Research Council of the Eye & ENT Hospital, Fudan University, Shanghai, China (No. KJ2008‐01).

## Conflict of Interest

The authors declare no conflict of interest.

## Author Contributions

X.K.Z., Y.H., J.Y.M., and D.Z. contributed equally to this work. L.M.L., L.T., and D.Z. designed the experiments. X.K.Z., Y.H., and J.Y.M. contributed to executing the described experiments. D.T., J.Z., Y.Y.J., and H.Q.L. were responsible for the bioinformatic analysis of the data. L.M.L., L.T., X.K.Z., and J.Y.M. analyzed the data. X.K.Z., C.D.H., and X.P.D. contributed to the preparation of this manuscript, and all authors aided in editing the final manuscript.

## Supporting information



Supporting Information

## Data Availability

The data that support the findings of this study are available from the corresponding author upon reasonable request.
