# Supplementary material for: Prolonged Survival of Neutrophils Induced by Tumor‐Derived G‐CSF/GM‐CSF Promotes Immunosuppression and Progression in Laryngeal Squamous Cell Carcinoma
Source: Adv Sci (Weinh). 2024 Oct 24;11(46):2400836. doi: 10.1002/advs.202400836 (PMC11633501; doi:10.1002/advs.202400836)
Supplement: Supplementary file 1 — Supporting Information [file ADVS-11-2400836-s001.docx]

**Supplementary Materials**

**CONTENTS**

**Supplementary Tables**

**Table S1.** Demographic and clinicopathological characteristics of the patients with LSCC.

**Table S2.** Antibodies and reagents used in this study.

**Table S3.** Univariate and multivariate analyses of factors associated with overall survival

**Table S4.** Multivariate analyses of factors associated with overall survival

**Supplementary Figures**

**Figure S1.** The correlations between neutrophil infiltration and clinicopathological parameters assessed by flow cytometry. (A) The number of CD66b^+^ neutrophils per 10^6^ cells was analyzed across different tissues in LSCC patients (n=50), with a focus on TNM staging (I+II versus III+IV). (B and C) Neutrophil proportion in CD45^+^ cells (A) or neutrophil number per 10^6^ cells (C) were assessed for correlations with clinicopathological parameters. Statistical analysis was conducted using one-way ANOVA, Mann-Whitney U tests, and Student’s t-test (***p˂0.001, **p˂0.01, *p˂0.05). ns, not significant; LSCC, laryngeal squamous cell carcinoma; ANT, adjacent normal tissue; PB, peripheral blood; LN, lymph node.

**Figure S2.** The correlations between neutrophil number per spot and clinicopathological parameters assessed by immunohistochemical staining. Statistical analysis was conducted using Mann-Whitney U tests and Student’s t-test (***p˂0.001, **p˂0.01, *p˂0.05). ns, not significant; LN, lymph node.

**Figure S3.** G-CSF/GM-CSF extend the lifespan of neutrophils in vitro. (A and B) Representative data and quantification of the percentage of viable (Annexin V^-^) cells on neutrophils cultured by 50% autologous NTCS or TTCS for 48h (A), or cultured by different concentrations of TTCS for 48h (B). (C) The expression of PD-L1, Siglec F, PD-L2, VISTA, and HLA-DR on neutrophils before and after TTCS stimulation. (D) The expression of several pro-inflammatory in head and neck cancer in TCGA database. (E and F) Representative data and quantification of the percentage of viable (Annexin V-) cells in neutrophils stimulated by IL1α, ILβ, IL8, IL33, CXCL5, M-CSF, TGFβ1, IFNα, CXCL1, or TNFα for 24h (E) or for 48h (F). (G) The percentage of viable (Annexin V^-^) cells in neutrophils stimulated by G-CSF (50ng/ml) or GM-CSF (50ng/ml) for 48h. (H)The percentage of viable (Annexin V^-^) cells in neutrophils cultured by 50% TTCS with or without G-CSF/GM-CSF neutralizing antibodies for 48h. Statistical analysis was conducted using one-way ANOVA, Mann-Whitney U tests, and Student’s t-test (***p˂0.001, **p˂0.01, *p˂0.05). ns, not significant; HNC, head and neck cancer; ANT, adjacent normal tissue; NTCS, Non-tumor tissue culture supernatant; TTCS, Tumor tissue culture supernatant.

**Figure S4.** G-CSF/GM-CSF regulate the apoptosis of neutrophils through the activation of PI3K/AKT signaling pathway. (A and B) The percentage of viable (Annexin V^-^) cells in neutrophils stimulated by 50% TTCS for 24h (A) and 48h (B), in the presence or absence of the inhibitors of JNK, JAK, ERK, MEK, STAT3, or mTOR. (C-E)Representative data and quantification of the percentage of viable (Annexin V-) cells in neutrophils cultured by 50% TTCS (C), or stimulated by G-CSF (D) or GM-CSF (E) for 48h with or without the inhibitors of PI3K, AKT, NFκB, or Mcl-1. (F)The percentage of viable (Annexin V-) cells in neutrophils cultured by different concentrations of TTCS. (G) Representative data and quantification of the proportion of live (Annexin V-) cells on neutrophils cultured by 50% TCCS with or without the presence of antibodies against G-CSF and/or GM-CSF for 48h. (H) The proportion of live (Annexin V-) cells on neutrophils cultured by 50% non-TCCS with or without the presence of G-CSF and/or GM-CSF. (I) The proportion of live (Annexin V-) cells on neutrophils cultured by 50% TCCS, in the presence or absence of the inhibitors of PI3K, AKT, NFκB or Mcl-1. Statistical analysis was conducted using one-way ANOVA, Mann-Whitney U tests, and Student’s t-test (***p˂0.001, **p˂0.01, *p˂0.05). ns, not significant; TTCS, Tumor tissue culture supernatant; TCCS, Tumor cell culture supernatant.

**Figure S5.** G-CSF/GM-CSF upregulate the proportion of viable (Annexin V^-^) CXCR4^+^ neutrophils. (A and B)The dynamic change of CXCR4 expression at a succession of moments in time on neutrophils cultured by 50% NTCS (A) or TTCS (B). (C)The proportion of CXCR4^+^ neutrophils were comparable between neutrophils cultured by complete RPMI-1640 medium, 50% NTCS or 50% TTCS. (D) Representative data and quantification of the percentage of viable (Annexin V^-^) CXCR4^+^ neutrophils cultured for 48h by 50% TTCS with or without antibodies against G-CSF and/or GM-CSF, or cultured by 50% NTCS with or without human G-CSF and/or GM-CSF. Statistical analysis was conducted using one-way ANOVA, Mann-Whitney U tests, and Student’s t-test (***p˂0.001, **p˂0.01, *p˂0.05). ns, not significant; NTCS, Non-tumor tissue culture supernatant; TTCS, Tumor tissue culture supernatant.

**Figure S6.** The phenotype of CXCR4^+^ neutrophils. (A-H) Representative images of PD-L2, VISTA, ROS, Arg1 and VEGF staining for CD66b^+^CD45^+^ neutrophils. The MFI of PD-L2(A), VISTA(C), ROS(E), Arg1(G) and VEGF (I) was compared between neutrophils derived from LSCC and ANT. The MFI of PD-L2(B), VISTA(D), ROS(F), Arg1(H) and VEGF (J) on neutrophils was compared between groups classified by the positive or negative PD-L1 expression. (I)The correlations between CXCR4 expression and the expression of PD-L2, VISTA, ROS or Arg1 on neutrophils. Statistical analysis was conducted using one-way ANOVA, Mann-Whitney U tests, Student’s t-test, and Spearman correlation analysis (***p˂0.001, **p˂0.01, *p˂0.05). ns, not significant; LSCC, laryngeal squamous cell carcinoma; ANT, adjacent normal tissue.

**Figure S7.** The relationship between CXCR4^+^ neutrophil infiltration and clinicopathological parameters assessed by immunofluorescence. CXCR4^+^ neutrophil proportion in total CD66b^+^ neutrophils (A) or the number of CXCR4^+^ neutrophils per spot (C) were assessed for correlations with clinicopathological parameters. Statistical analysis was conducted using Mann-Whitney U tests and Student’s t-test (***p˂0.001, **p˂0.01, *p˂0.05). ns, not significant; LN, lymph node.

**Figure S8.** The correlation between viable (Annexin^-^) neutrophil infiltration and the exhaustion of CD8^+^ T cells in LSCC tissue assessed by flow cytometry (A-C). The correlation between CXCR4^+^ neutrophil infiltration and the exhaustion of CD8^+^ T cells in LSCC tissue assessed by flow cytometry (D-E). Statistical analysis was conducted using Spearman correlation analysis (**p˂0.01, *p˂0.05).

| **Table S1.** Demographic and clinicopathological characteristics of the patients with LSCC | | |
| --- | --- | --- |
| **Variables** | **Cohort 1** | **Cohort 2** |
| **Total, n** | **61** | **50** |
| **Age at diagnosis** |  |  |
| ≤60 | 22(36.2%) | 21(42.0%) |
| >60 | 39(63.9%) | 29(58.0%) |
| **Smoking history, n (%)** |  |  |
| No | 18(29.5%) | 14(28.0%) |
| Yes | 43(70.5%) | 36(72.0%) |
| **Drinking history, n (%)** |  |  |
| No | 33(54.1%) | 22(44.0%) |
| Yes | 28(45.9%) | 28(56.0%) |
| **Diabetes, n (%)** |  |  |
| No | 53(86.9%) | 30(60.0%) |
| Yes | 8(13.1%) | 20(40.0%) |
| **Hypertension, n (%)** |  |  |
| ≤3.5cm | 44(72.1%) | 31(62.0%) |
| >3.5cm | 17(27.9%) | 19(38.0%) |
| **T stage, n (%)** |  |  |
| T1-2 | 17(27.9%) | 21(42.0%) |
| T3-4 | 44(72.1%) | 29(58.0%) |
| **N stage, n (%)** |  |  |
| N0 | 34(55.7%) | 30(60.0%) |
| N1-2 | 27(44.3%) | 20(40.0%) |
| **TNM stage, n (%)** |  |  |
| I-II | 14(23.0%) | 21(42.0%) |
| III-VI | 47(77.0%) | 29(58.0%) |
| **Tumor size , n (%)** |  |  |
| ≤3.5cm | 37(60.7%) | 26(52.0%) |
| >3.5cm | 24(39.3%) | 24(48.0%) |
| **Pathological LN fusion** |  |  |
| No | 54(88.5%) | 40(80.0%) |
| Yes | 7(11.5%) | 10(20.0%) |
| **Vascular invasion** |  |  |
| No | 51(83.6%) | 39(78.0%) |
| Yes | 10(16.4%) | 11(22.0%) |
| Abbreviation: LSCC, Laryngeal squamous cell carcinoma; LNs, Lymph nodes | | |

| **Table S2.** Antibodies and reagents used in this study. | |
| --- | --- |
| **Antibodies and reagents** | **Manufacturers** |
| **Antibodies for immunohistochemical and immunofluorescence staining** |  |
| Anti-CD66b antibody | Abcam |
| Anti-Ki67 antibody | Abcam |
| Anti-PCNA antibody [PC10] | Abcam |
| Human GM-CSF Antibody | R&D Systems |
| Human G-CSF Antibody | Santa Cruz Biotechnology |
| Human Ckpan Antibody | Abcam |
| Human CXCR4 Antibody | R&D Systems |
| CD8 Monoclonal Antibody | Invitrogen Antibodies |
| Goat Anti-Rabbit IgG H&L (Alexa Fluor® 488) | Abcam |
| Goat Anti-Mouse IgG H&L (Cy3 ®) | Abcam |
| DAB kit | Abcam |
| Goat F(ab) Anti-Rabbit/Mouse IgG H&L (Biotin) | Abcam |
| **Antibodies for western blot** |  |
| Phospho-PI3 Kinase p85 (Tyr458)/p55 (Tyr199) Antibody | CST |
| PI3 Kinase p110α (C73F8) Rabbit mAb | CST |
| Phospho-Akt (Ser473) (D9E) XP® Rabbit mAb | CST |
| Akt (pan) (C67E7) Rabbit mAb | CST |
| Mcl-1 (D35A5) Rabbit mAb | CST |
| Cleaved Caspase-3 (Asp175) Antibody | CST |
| Caspase-3 Antibody | CST |
| Anti-GAPDH antibody | CST |
| Anti-rabbit IgG, HRP-linked Antibody | CST |
| **ELISA kits** |  |
| Human GM-CSF ELISA Kit | Lianke Biotechnology |
| Human G-CSF ELISA Kit | Lianke Biotechnology |
| **Antibodies for neutralizing and blocking** |  |
| Human GM-CSF Antibody | Abcam |
| Human G-CSF Antibody | R&D Systems |
| Monoclonal Mouse IgG1 | Abcam |
| VU661013, Mcl-1 inhibitor | Selleck |
| Tanzisertib, JNK inhibitor | Selleck |
| Ruxolitinib, JAK inhibitor | Selleck |
| SCH772984, ERK inhibitor | Selleck |
| RDEA119, MEK inhibitor | Selleck |
| Ochromycinone, STAT inhibitor | Selleck |
| KU-0063794, mTOR inhibitor | Selleck |
| Wortmannin, PI3K inhibitor | Selleck |
| MK-2206 2HCl, AKT inhibitor | Selleck |
| BAY 11-7082, NFkB inhibitor | Selleck |
| **Antibodies for flow cytometry** |  |
| Anti-CD66b antibody APC | Biolegend |
| Anti-CXCR4 antibody PerCP-Cy5.5 | Biolegend |
| Anti-CXCR2 antibody PE | Biolegend |
| Anti-CD45 antibody BV711 | Biolegend |
| Anti-CD62L antibody PE-Cy7 | BD Pharmingen |
| Anti-Siglec F(CD170) antibody FITC | Biolegend |
| Anti-HLA-DR antibody BV421 | Biolegend |
| Anti-PD-L1 antibody BV605 | Biolegend |
| Anti-CD11b antibody APC-Cy7 | Biolegend |
| Anti-PD-L2 antibody PE | Biolegend |
| Anti-VISTA antibody BV421 | Biolegend |
| Anti-Active anti-Caspase3 antibody BV605 | BD Pharmingen |
| Anti-ARG1 antibody PE | BD Pharmingen |
| Anti-ROS antibody FITC | BD Pharmingen |
| Annexin V-FICT/PI | Yeasen Biotechnology |
| Carboxylfluorescein succinimidyl ester (CFSE) | eBioscience |
| **Other antibodies and reagents** |  |
| All recombinant cytokines and chemokines | PeproTech |
| EasySep™ HLA Chimerism Whole Blood CD66b Positive Selection Kit | Stemcell |
| EasySep™ HLA Chimerism Whole Blood CD8 Positive Selection Kit | Stemcell |
| EasySep™ Direct Human Monocyte Isolation Kit | Stemcell |

| **Table S3.** Univariate and multivariate analyses of factors associated with overall survival | | | | | |
| --- | --- | --- | --- | --- | --- |
|  | **Univariate** | |  | **Multivariate** | |
|  | **HR(95% CI)** | **P value** |  | **HR(95% CI)** | **P value** |
| **Age** |  | 0.032 |  |  | 0.087 |
| ≤60 | Ref. |  |  | Ref. |  |
| >60 | 3.856(1.122-13.246) |  |  | 3.274(0.841-12.741) |  |
| **Smoking history, n (%)** |  | 0.021 |  |  | 0.047 |
| No | Ref. |  |  | Ref. |  |
| Yes | 5.644(1.291-24.673) |  |  | 4.683(1.023-21.435) |  |
| **Drinking history, n (%)** |  | 0.205 |  |  |  |
| No | Ref. |  |  |  |  |
| Yes | 1.810(0.724-4.529) |  |  |  |  |
| **Diabetes, n (%)** |  | 0.73 |  |  |  |
| No | Ref. |  |  |  |  |
| Yes | 0.773(0.178-3.347) |  |  |  |  |
| **Hypertension, n (%)** |  | 0.125 |  |  |  |
| ≤3.5cm | Ref. |  |  |  |  |
| >3.5cm | 0.379(0.110-1.310) |  |  |  |  |
| **T stage, n (%)** |  | 0.063 |  |  |  |
| T1-2 | Ref. |  |  |  |  |
| T3-4 | 4.034(0.927-17.551) |  |  |  |  |
| **N stage, n (%)** |  | 0.027 |  | Ref. | 0.332 |
| N0 | Ref. |  |  |  |  |
| N1-2 | 3.017(1.132-8.038) |  |  | 1.731(0.571-5.243) |  |
| **TNM stage, n (%)** |  | 0.05 |  | Ref. | 0.228 |
| I-II | Ref. |  |  |  |  |
| III-VI | 7.486(0.996-56.257) |  |  | 3.863(0.428-34.845) |  |
| **Tumor size , n (%)** |  | 0.171 |  |  |  |
| ≤3.5cm | Ref. |  |  |  |  |
| >3.5cm | 1.903(0.758-4.778) |  |  |  |  |
| **Pathological LN fusion** |  | 0.182 |  |  |  |
| No | Ref. |  |  |  |  |
| Yes | 2.363(0.668-8.357) |  |  |  |  |
| **Vascular invasion** |  | 0.691 |  |  |  |
| No | Ref. |  |  |  |  |
| Yes | 1.286(0.372-4.451) |  |  |  |  |
| **Number of TANs** |  | 0.015 |  | Ref. | 0.108 |
| <50 | Ref. |  |  |  |  |
| >50 | 4.637(1.346-15.976) |  |  | 2.949(0.790-11.013) |  |
| Abbreviation: LSCC, Laryngeal squamous cell carcinoma; LNs, Lymph nodes; TANs, tumor-associated neutrophils | | | | | |

| **Table S4.** Multivariate analyses of factors associated with overall survival | | | | | |
| --- | --- | --- | --- | --- | --- |
|  | **HR(95% CI)** | **P value** |  | **HR(95% CI)** | **P value** |
| **Age at diagnosis** |  | 0.011 |  |  | 0.02 |
| ≤60 |  |  |  |  |  |
| >60 | 5.314(1.475-19.146) |  |  | 4.407(1.260-15.420) |  |
| **Smoking history, n (%)** |  | 0.042 |  |  | 0.061 |
| No |  |  |  |  |  |
| Yes | 4.868(1.058-22.399) |  |  | 4.271(0.933-19.557) |  |
| **N stage, n (%)** |  | 0.715 |  |  | 0.999 |
| N0 |  |  |  |  |  |
| N1-2 | 1.223(0.416-3.594) |  |  | 0.999(0.341-2.927) |  |
| **TNM stage, n (%)** |  | 0.299 |  |  | 0.296 |
| I-II |  |  |  |  |  |
| III-VI | 3.364(0.342-33.136) |  |  | 3.270(0.355-30.125) |  |
| **Number of CXCR4^+^ TANs** |  | 0.201 |  |  |  |
| <10 |  |  |  |  |  |
| >10 | 2.257(0.647-7.872) |  |  |  |  |
| **Percentage of CXCR4^+^ TANs** |  |  |  |  | 0.039 |
| <20% |  |  |  |  |  |
| >20% |  |  |  | 4.149(1.076-15.995) |  |

**Figure S1**


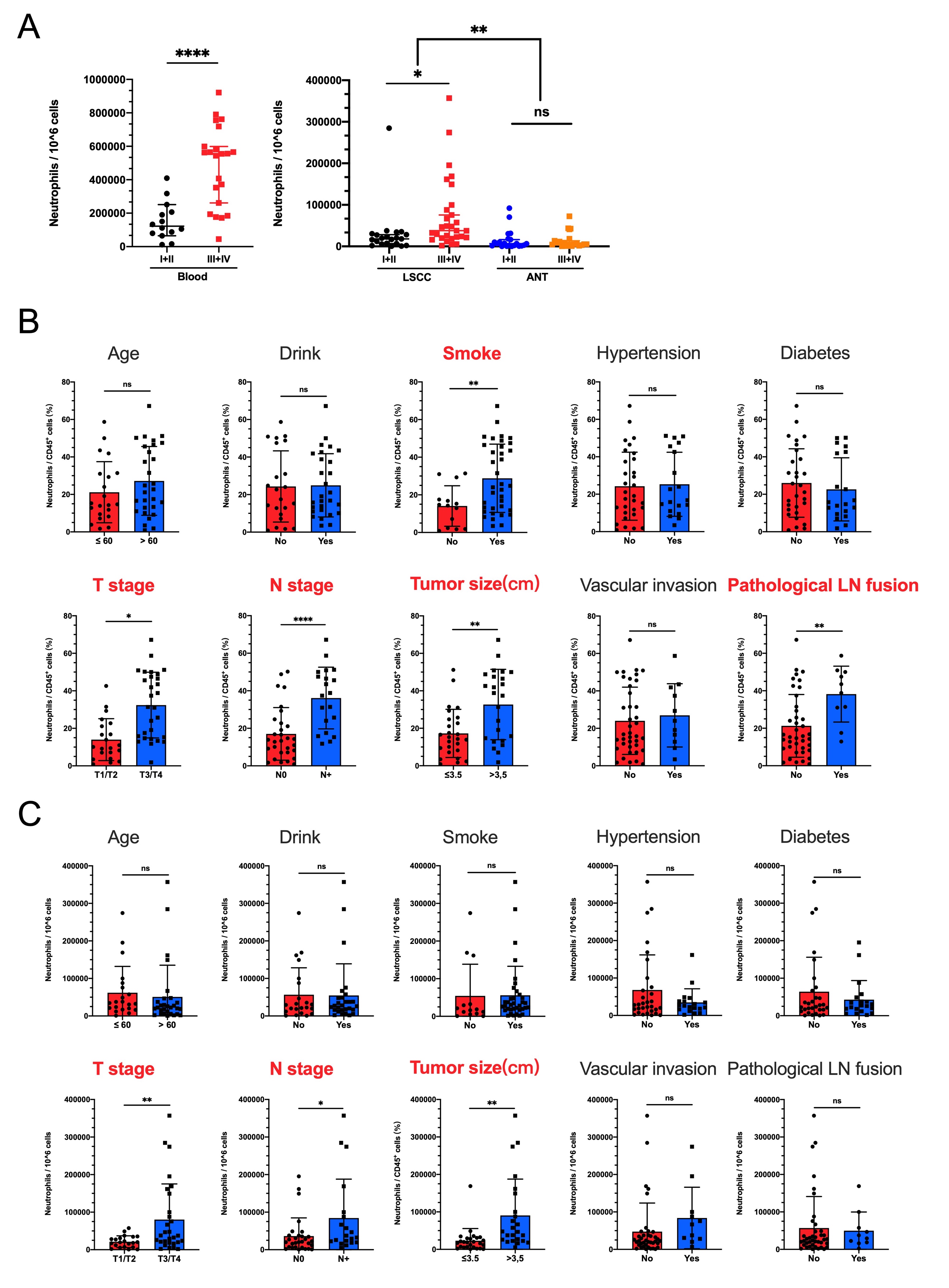


**Figure S1.** The correlations between neutrophil infiltration and clinicopathological parameters assessed by flow cytometry. (A) The number of CD66b^+^ neutrophils per 10^6^ cells was analyzed across different tissues in LSCC patients (n=50), with a focus on TNM staging (I+II versus III+IV). (B and C) Neutrophil proportion in CD45^+^ cells (A) or neutrophil number per 10^6^ cells (C) were assessed for correlations with clinicopathological parameters. Statistical analysis was conducted using one-way ANOVA, Mann-Whitney U tests, and Student’s t-test (***p˂0.001, **p˂0.01, *p˂0.05). ns, not significant; LSCC, laryngeal squamous cell carcinoma; ANT, adjacent normal tissue; PB, peripheral blood; LN, lymph node.

**Figure S2**


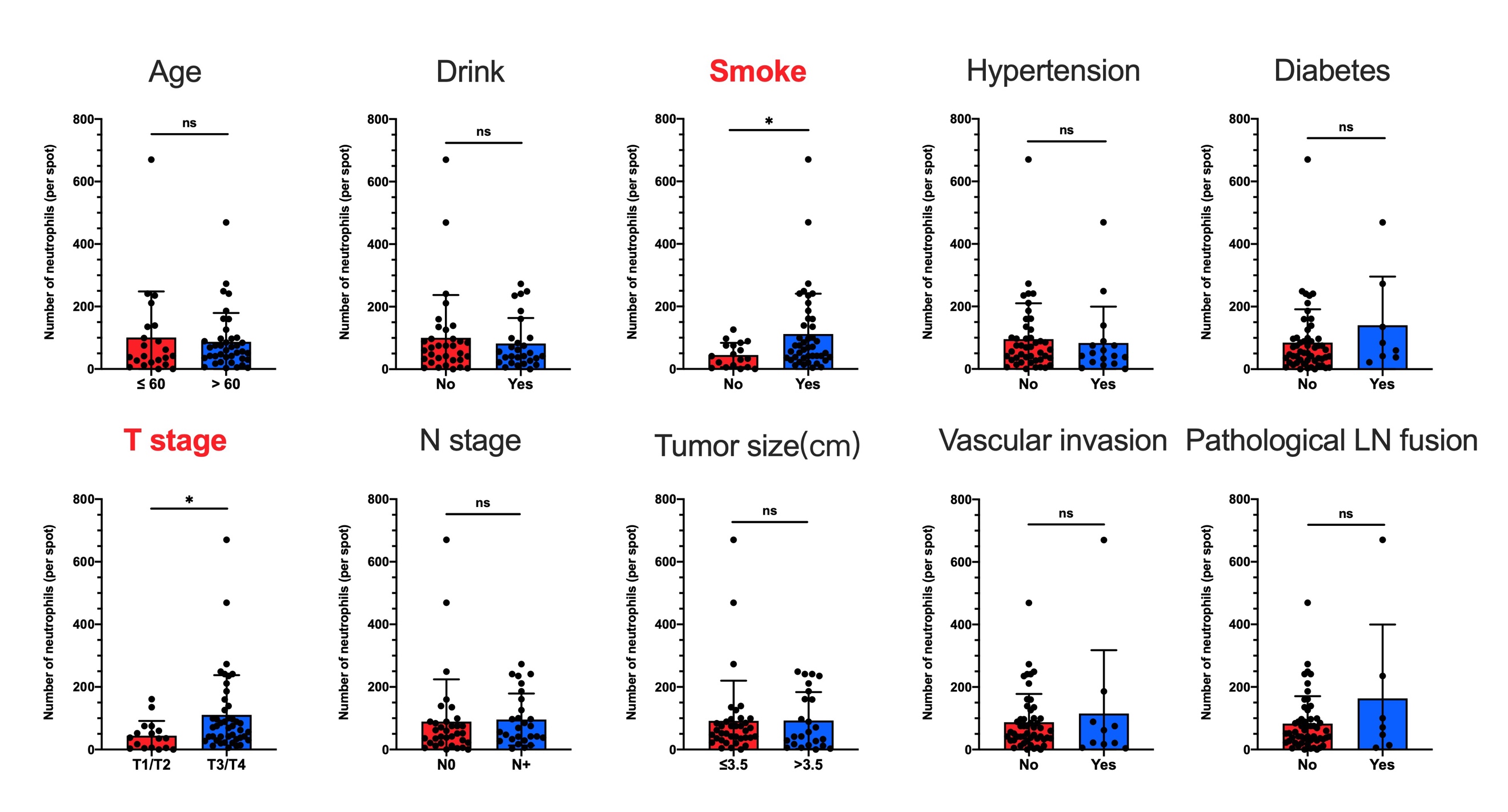


**Figure S2.** The correlations between neutrophil number per spot and clinicopathological parameters assessed by immunohistochemical staining. Statistical analysis was conducted using Mann-Whitney U tests and Student’s t-test (***p˂0.001, **p˂0.01, *p˂0.05). ns, not significant; LN, lymph node.

**Figure S3**

**Figure S3.** G-CSF/GM-CSF extend the lifespan of neutrophils in vitro. (A and B) Representative data and quantification of the percentage of viable (Annexin V^-^) cells on neutrophils cultured by 50% autologous NTCS or TTCS for 48h (A), or cultured by different concentrations of TTCS for 48h (B). (C) The expression of PD-L1, Siglec F, PD-L2, VISTA, and HLA-DR on neutrophils before and after TTCS stimulation. (D) The expression of several pro-inflammatory in head and neck cancer in TCGA database. (E and F) Representative data and quantification of the percentage of viable (Annexin V-) cells in neutrophils stimulated by IL1α, ILβ, IL8, IL33, CXCL5, M-CSF, TGFβ1, IFNα, CXCL1, or TNFα for 24h (E) or for 48h (F). (G) The percentage of viable (Annexin V^-^) cells in neutrophils stimulated by G-CSF (50ng/ml) or GM-CSF (50ng/ml) for 48h. (H)The percentage of viable (Annexin V^-^) cells in neutrophils cultured by 50% TTCS with or without G-CSF/GM-CSF neutralizing antibodies for 48h. Statistical analysis was conducted using one-way ANOVA, Mann-Whitney U tests, and Student’s t-test (***p˂0.001, **p˂0.01, *p˂0.05). ns, not significant; HNC, head and neck cancer; ANT, adjacent normal tissue; NTCS, Non-tumor tissue culture supernatant; TTCS, Tumor tissue culture supernatant.

**Figure S4**


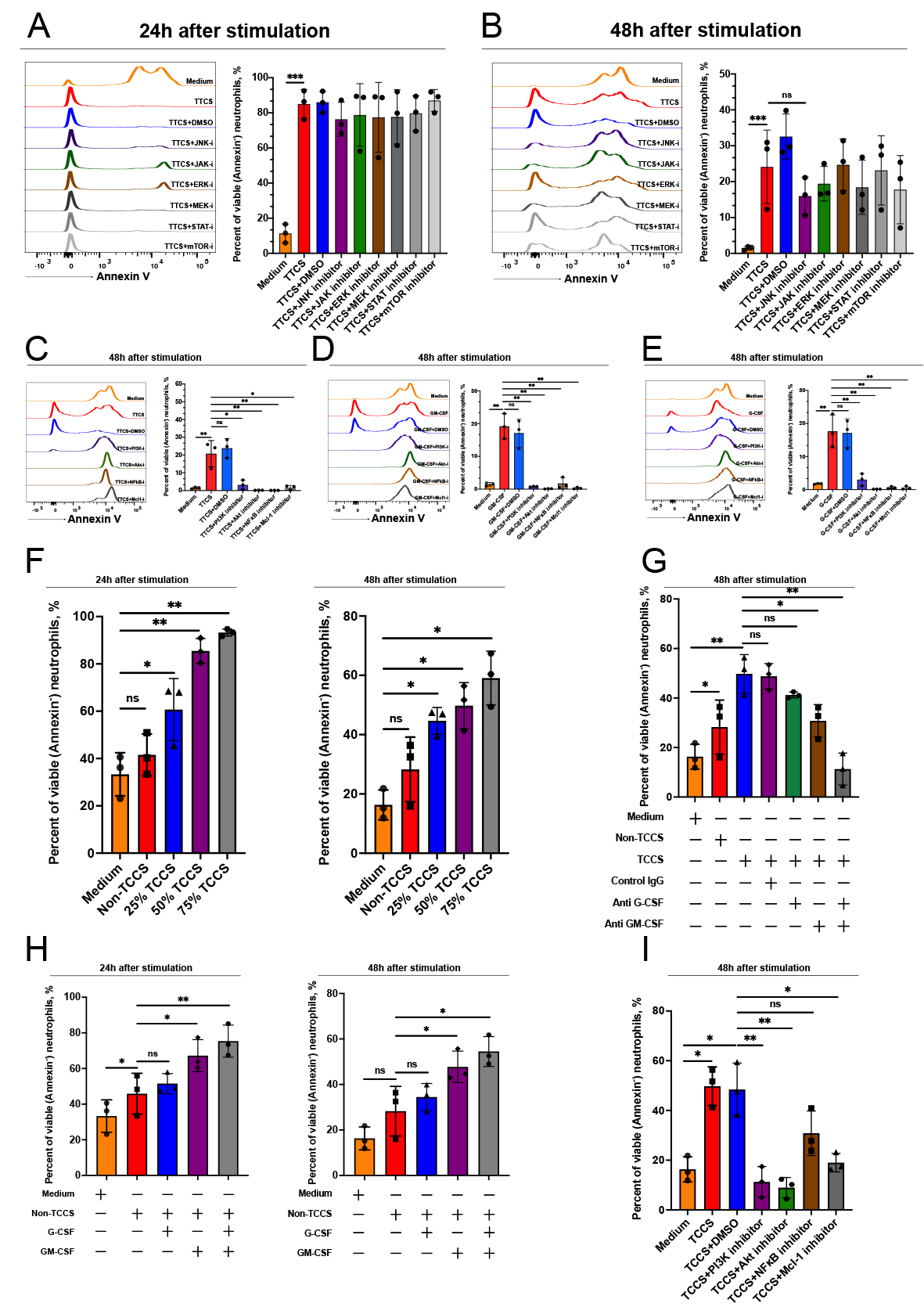


**Figure S4.** G-CSF/GM-CSF regulate the apoptosis of neutrophils through the activation of PI3K/AKT signaling pathway. (A and B) The percentage of viable (Annexin V^-^) cells in neutrophils stimulated by 50% TTCS for 24h (A) and 48h (B), in the presence or absence of the inhibitors of JNK, JAK, ERK, MEK, STAT3, or mTOR. (C-E)Representative data and quantification of the percentage of viable (Annexin V-) cells in neutrophils cultured by 50% TTCS (C), or stimulated by G-CSF (D) or GM-CSF (E) for 48h with or without the inhibitors of PI3K, AKT, NFκB, or Mcl-1. (F)The percentage of viable (Annexin V-) cells in neutrophils cultured by different concentrations of TTCS. (G) Representative data and quantification of the proportion of live (Annexin V-) cells on neutrophils cultured by 50% TCCS with or without the presence of antibodies against G-CSF and/or GM-CSF for 48h. (H) The proportion of live (Annexin V-) cells on neutrophils cultured by 50% non-TCCS with or without the presence of G-CSF and/or GM-CSF. (I) The proportion of live (Annexin V-) cells on neutrophils cultured by 50% TCCS, in the presence or absence of the inhibitors of PI3K, AKT, NFκB or Mcl-1. Statistical analysis was conducted using one-way ANOVA, Mann-Whitney U tests, and Student’s t-test (***p˂0.001, **p˂0.01, *p˂0.05). ns, not significant; TTCS, Tumor tissue culture supernatant; TCCS, Tumor cell culture supernatant.

**Figure S5**


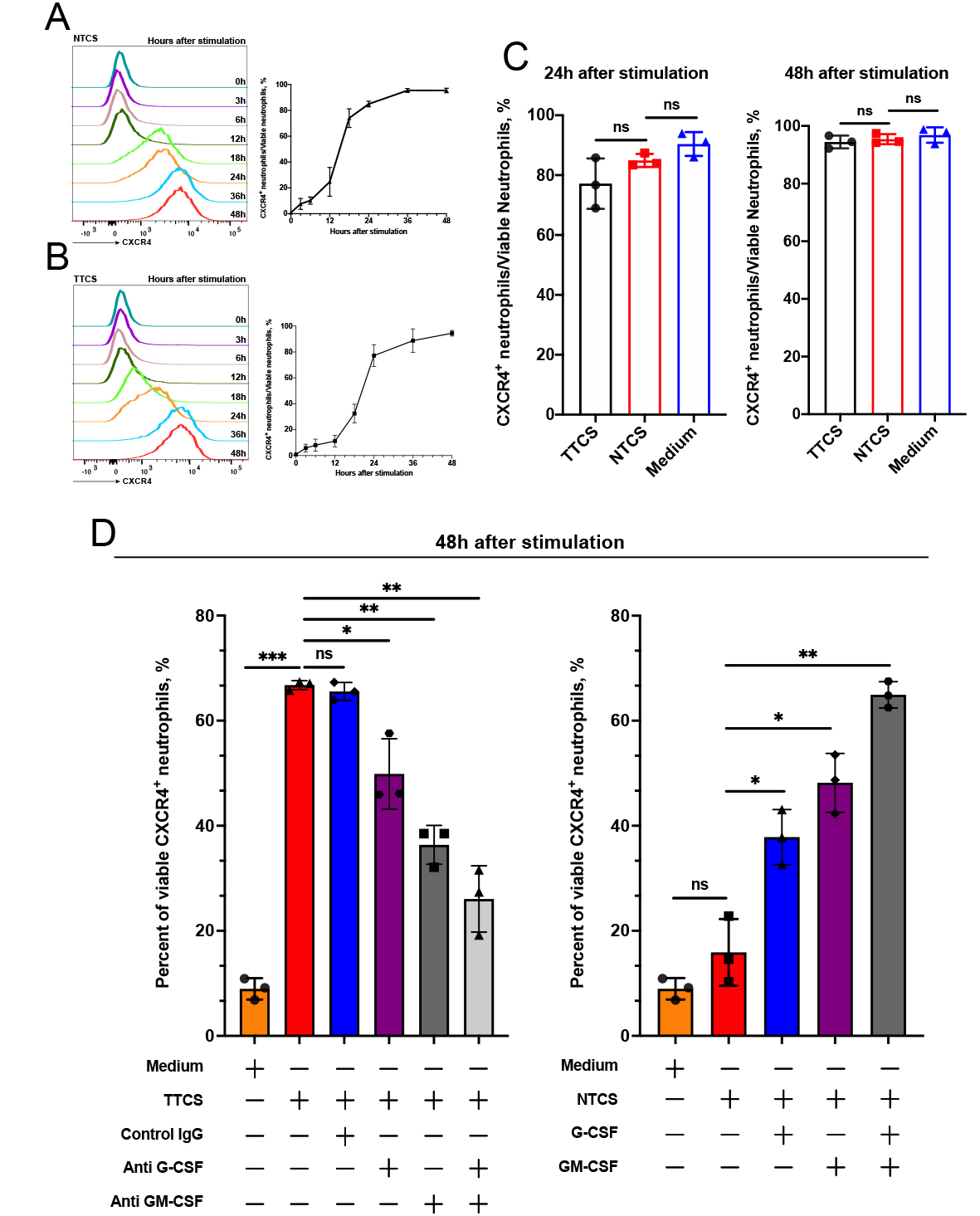


**Figure S5.** G-CSF/GM-CSF upregulate the proportion of viable (Annexin V^-^) CXCR4^+^ neutrophils. (A and B)The dynamic change of CXCR4 expression at a succession of moments in time on neutrophils cultured by 50% NTCS (A) or TTCS (B). (C)The proportion of CXCR4^+^ neutrophils were comparable between neutrophils cultured by complete RPMI-1640 medium, 50% NTCS or 50% TTCS. (D) Representative data and quantification of the percentage of viable (Annexin V^-^) CXCR4^+^ neutrophils cultured for 48h by 50% TTCS with or without antibodies against G-CSF and/or GM-CSF, or cultured by 50% NTCS with or without human G-CSF and/or GM-CSF. Statistical analysis was conducted using one-way ANOVA, Mann-Whitney U tests, and Student’s t-test (***p˂0.001, **p˂0.01, *p˂0.05). ns, not significant; NTCS, Non-tumor tissue culture supernatant; TTCS, Tumor tissue culture supernatant.

**Figure S6**


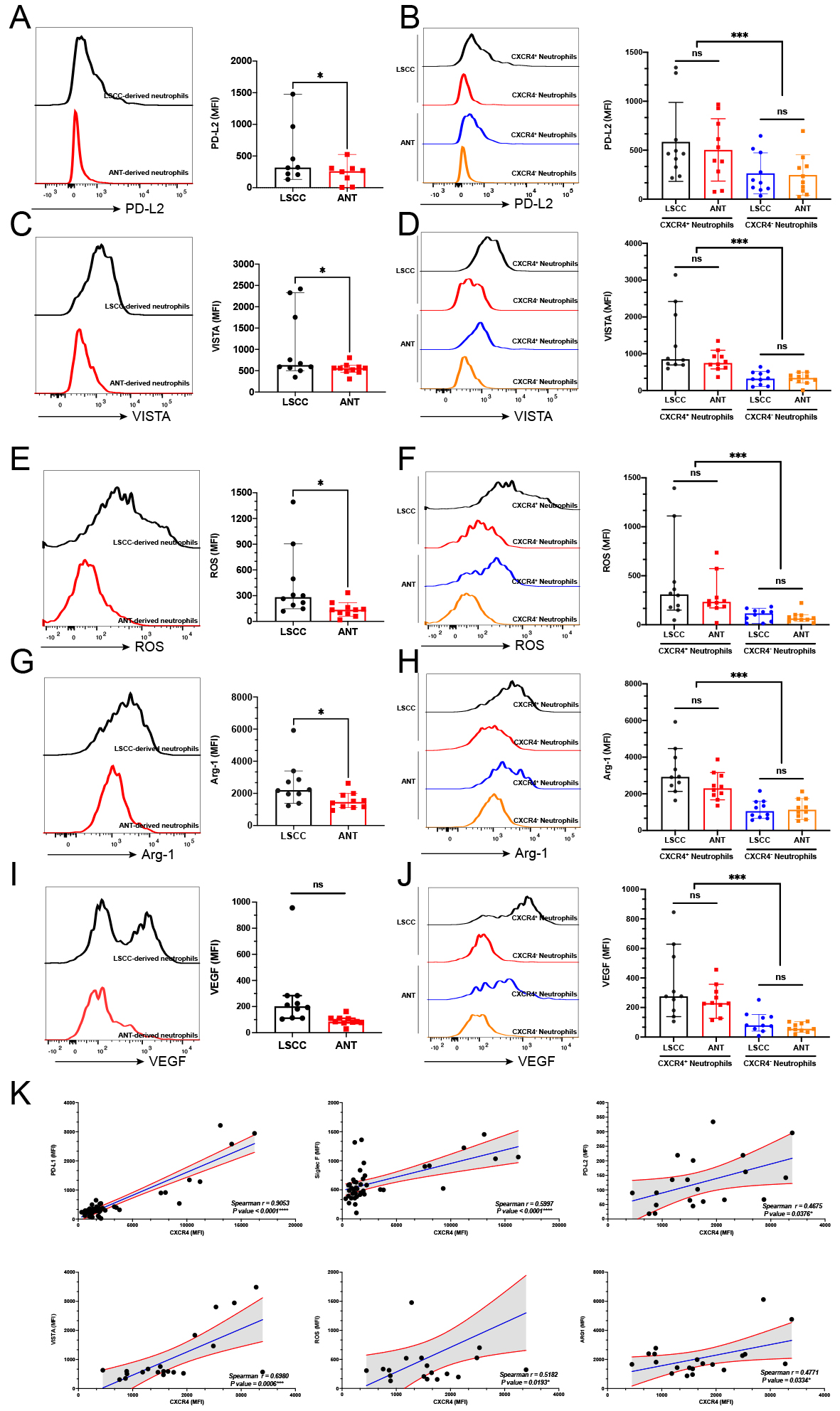


**Figure S6.** The phenotype of CXCR4^+^ neutrophils. (A-H) Representative images of PD-L2, VISTA, ROS, Arg1 and VEGF staining for CD66b^+^CD45^+^ neutrophils. The MFI of PD-L2(A), VISTA(C), ROS(E), Arg1(G) and VEGF (I) was compared between neutrophils derived from LSCC and ANT. The MFI of PD-L2(B), VISTA(D), ROS(F), Arg1(H) and VEGF (J) on neutrophils was compared between groups classified by the positive or negative PD-L1 expression. (I)The correlations between CXCR4 expression and the expression of PD-L2, VISTA, ROS or Arg1 on neutrophils. Statistical analysis was conducted using one-way ANOVA, Mann-Whitney U tests, Student’s t-test, and Spearman correlation analysis (***p˂0.001, **p˂0.01, *p˂0.05). ns, not significant; LSCC, laryngeal squamous cell carcinoma; ANT, adjacent normal tissue.

**Figure S7**


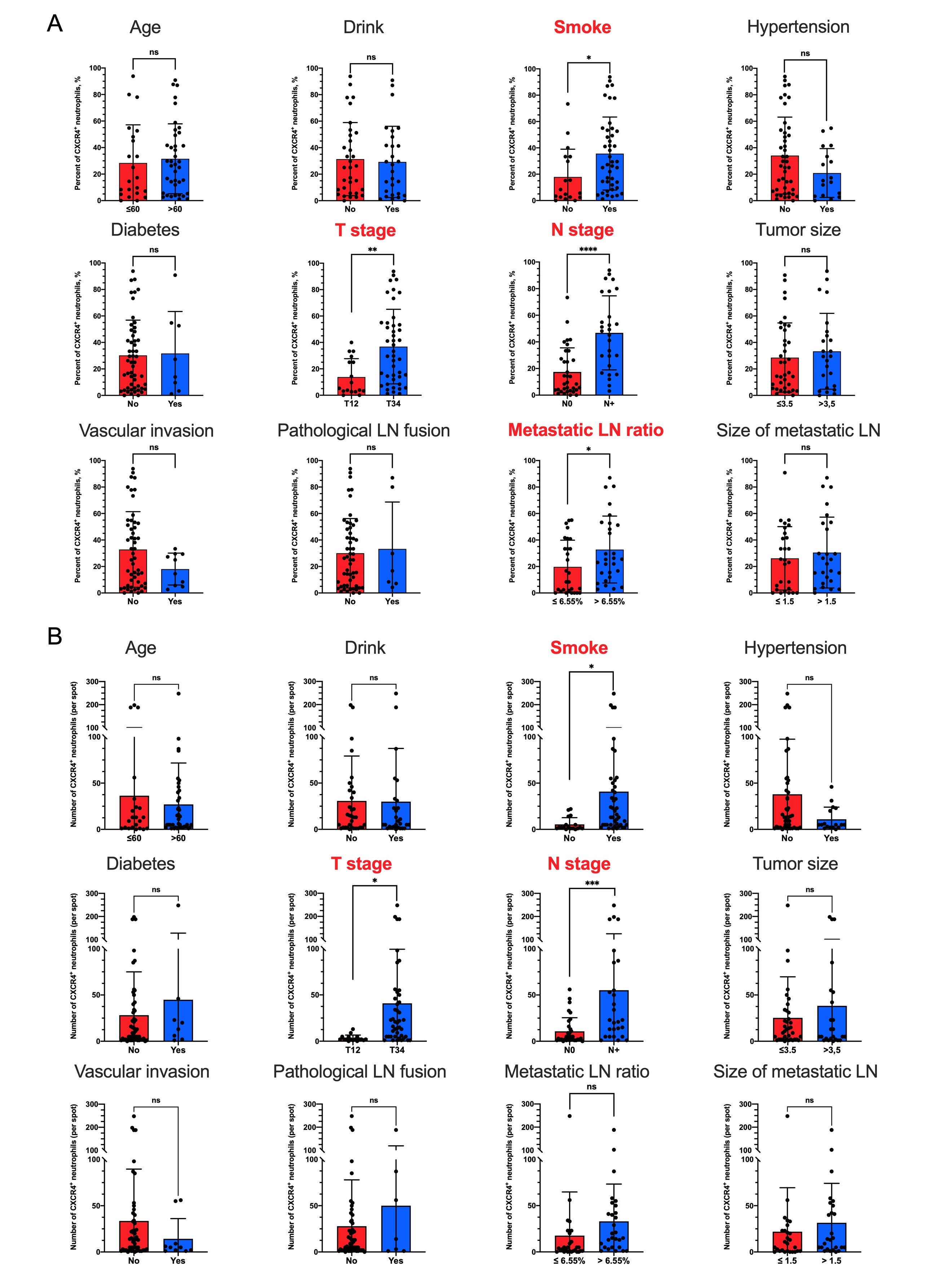


**Figure S7.** The relationship between CXCR4^+^ neutrophil infiltration and clinicopathological parameters assessed by immunofluorescence. CXCR4^+^ neutrophil proportion in total CD66b^+^ neutrophils (A) or the number of CXCR4^+^ neutrophils per spot (C) were assessed for correlations with clinicopathological parameters. Statistical analysis was conducted using Mann-Whitney U tests and Student’s t-test (***p˂0.001, **p˂0.01, *p˂0.05). ns, not significant; LN, lymph node.

**Figure S8**


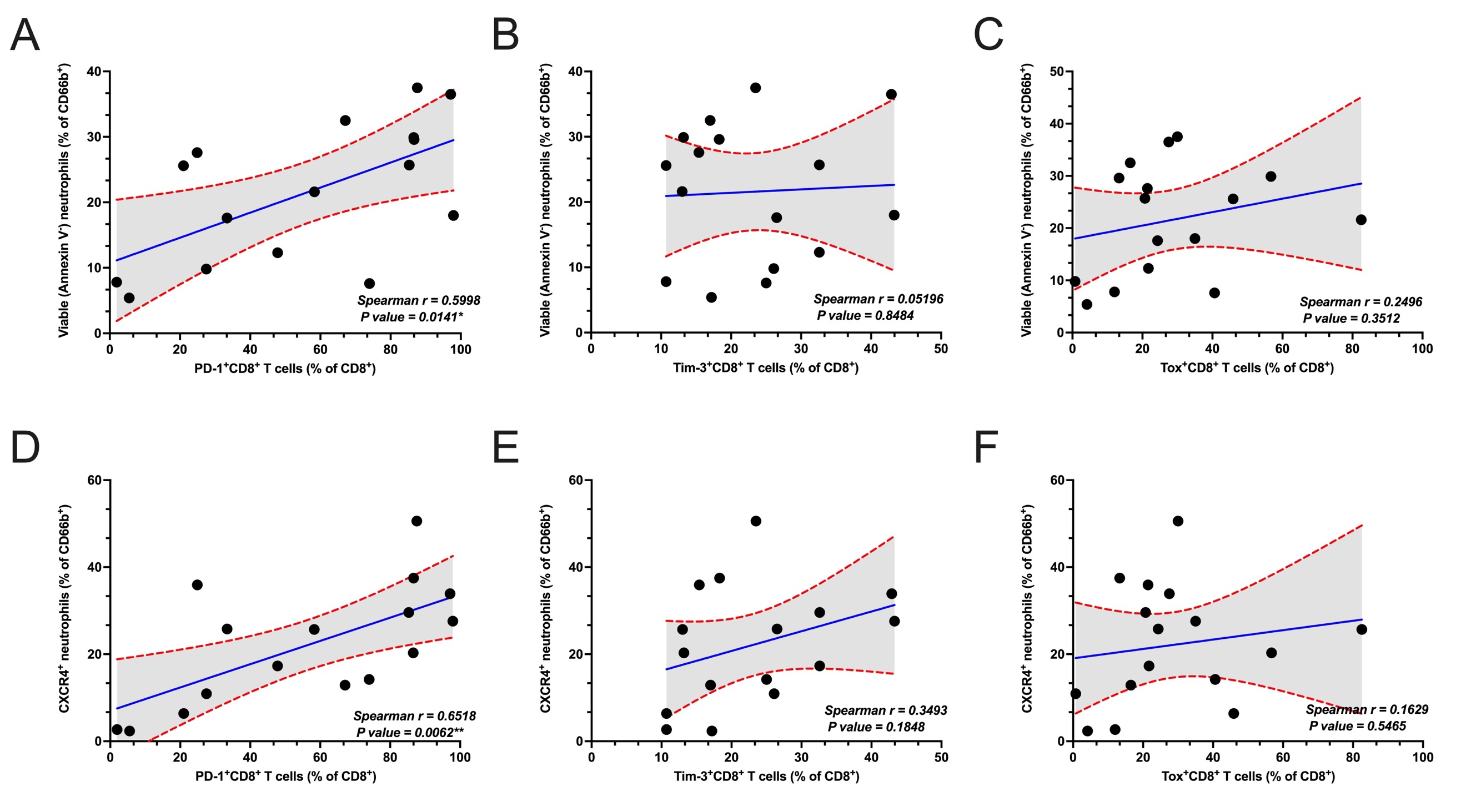


**Figure S8.** The correlation between viable (Annexin^-^) neutrophil infiltration and the exhaustion of CD8^+^ T cells in LSCC tissue assessed by flow cytometry (A-C). The correlation between CXCR4^+^ neutrophil infiltration and the exhaustion of CD8^+^ T cells in LSCC tissue assessed by flow cytometry (D-E). Statistical analysis was conducted using Spearman correlation analysis (**p˂0.01, *p˂0.05).
